# Amino Acid Metabolism in Health and Disease

**DOI:** 10.1002/mco2.70930

**Published:** 2026-08-28

**Authors:** Zhiwei Su, Jinyu Liu, Xiaoliang Deng, Yanqun Luo, Haiping Xue, Lei Wang, Lianjun Xing, Tao Wu

**Affiliations:** ^1^ Department II of Digestive Diseases, Longhua Hospital Shanghai University of Traditional Chinese Medicine Shanghai China; ^2^ Institute of Interdisciplinary Integrative Medicine Research Shanghai University of Traditional Chinese Medicine Shanghai China; ^3^ Industrial Development Center Shanghai University of Traditional Chinese Medicine Shanghai China; ^4^ Department of Hepatology, Longhua Hospital Shanghai University of Traditional Chinese Medicine Shanghai China

**Keywords:** amino acid metabolism, branched‐chain amino acids, clinical translation, epigenetic regulation, gut microbiota–amino acid axis, metabolic reprogramming, one‐carbon metabolism, redox homeostasis

## Abstract

Metabolic reprogramming is a central feature of many human diseases, and therapies that target altered metabolic dependencies are moving from concept to clinical testing. Amino acid homeostasis links essential and nonessential amino acid supply with branched‐chain amino acid (BCAA) catabolism, one‐carbon metabolism, mechanistic target of rapamycin complex 1 (mTORC1)/general control nonderepressible 2 (GCN2) nutrient sensing, glutathione‐dependent redox control, epigenetic regulation, and the gut microbiota–amino acid axis. When this network is disturbed, amino acid flux can contribute to disease initiation and progression rather than simply mirroring established pathology. This review synthesizes how amino acid metabolism supports the nervous, cardiovascular, digestive, metabolic–endocrine, immune, skeletal, urinary and reproductive systems, as well as malignant and inherited metabolic disorders. We also examine how pathway‐level disturbances converge on excitotoxicity, endothelial dysfunction, insulin resistance, inflammation, fibrosis, immune escape, toxic metabolite accumulation, and impaired fertility. Rather than catalog isolated findings, we emphasize unifying principles and unresolved controversies, including the context‐dependent effects of BCAA signaling, the causal versus biomarker status of circulating amino acid signatures, host–gut microbiome crosstalk, and the therapeutic window of dietary, enzymatic, transporter‐targeted, and microbiota‐based interventions. Finally, we assess clinical translation, drawing lessons from late‐stage trial failures and emerging strategies with realistic potential for precision metabolic therapy.

## Graphical abstract

This graphical abstract delineates the multifaceted role of amino acid metabolism in health and disease. It illustrates how amino acids sustain physiological homeostasis across the liver, kidney, brain, heart, intestine, muscle, skeleton, and immune system. Key sensing pathways, including mTORC1 and GCN2, coordinate metabolic mediators such as SAM, acetyl‐CoA, α‐ketoglutarate (α‐KG), and glutathione, thereby regulating epigenetic remodeling and redox defense. The abstract summarizes how metabolic dysregulation contributes to tumor progression, immune evasion, insulin resistance, neuroexcitation, atherosclerosis, intestinal barrier impairment, and renal fibrosis. It also highlights the gut microbiota–amino acid axis through indoles, short‐chain fatty acids, and polyamines. Finally, it presents therapeutic strategies, including targeted inhibitors, dietary restriction, supplementation, microbiota modulation, and nanoparticle‐based delivery.

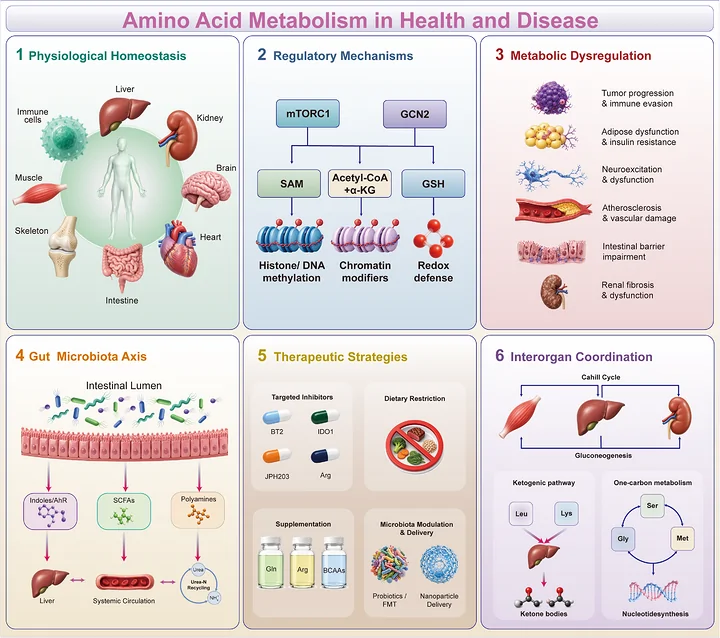

## 1 Introduction

Metabolic reprogramming is increasingly viewed as a driver of disease biology, not only a consequence of tissue injury. Altered nutrient use contributes to malignancy, chronic metabolic disease, immune dysfunction, and degenerative disorders [1, 2]. This has made metabolic dependencies attractive therapeutic entry points, although the gain in tissue selectivity must be balanced against toxicity in normal organs [3]. Within this landscape, amino acid homeostasis occupies a distinctive position because it connects protein synthesis, nitrogen handling, redox control, nutrient sensing, and interorgan exchange [4, 5, 6].

Twenty proteinogenic amino acids form the core vocabulary of mammalian metabolism. Nine essential amino acids (EAAs) cannot be synthesized de novo in humans and therefore depend on dietary supply [7]. Beyond their structural role in proteins, amino acids provide precursors for nucleotides, neurotransmitters, hormones, porphyrins, and glutathione (GSH), a major antioxidant system [8, 9, 10]. They also function as nutrient signals; leucine and other amino acids, for example, regulate mechanistic target of rapamycin complex 1 (mTORC1), which coordinates growth, proliferation, and autophagy [11]. At the whole‐body level, amino acid balance depends on organ specialization: the liver mediates catabolism and interconversion, skeletal muscle is a major site of branched‐chain amino acid (BCAA) breakdown, and the intestine and kidneys control absorption, recycling, and nitrogen excretion [12, 13, 14, 15].

The effects of amino acids are strongly context dependent. In immune cells, substrate availability shapes maturation and effector function; glutamine (Gln) and arginine (Arg) support pathogen clearance, whereas tryptophan (Trp) metabolism helps maintain immune tolerance [16, 17]. Amino acid‐derived methyl donors and acetyl‐coenzyme A (acetyl‐CoA) also connect nutrient state to DNA and histone modification [18]. At the organismal scale, methionine (Met) or BCAA restriction has been associated with delayed aging phenotypes in experimental systems, linking amino acid availability to healthspan while underscoring the need for context‐specific interpretation [19, 20].

Disease settings reveal the same duality. Oncogenic programs driven by KRAS or MYC can create dependencies on Gln or serine (Ser), supporting proliferation and immune evasion [21, 22, 23, 24]. Dietary or enzymatic depletion of selected amino acids can exploit these vulnerabilities in defined contexts [25, 26]. In metabolic disease, elevated circulating branched‐chain and aromatic amino acids predict insulin resistance (IR) across obesity, Type 2 diabetes (T2D), and fatty liver disease [27]. Amino acid imbalance is also implicated in cardiovascular, autoimmune, neurodegenerative, and inherited metabolic disorders, although causal strength differs across disease groups [28, 29, 30, 31].

Several critical gaps remain in this field. Cell‐type‐specific regulation of amino acid flux remains incompletely resolved, and bidirectional host–microbiota exchange is often inferred from circulating metabolites rather than measured directly. Clinical translation faces similar obstacles, including weak predictive biomarkers, limited patient stratification, and toxicity from perturbing pathways shared by diseased and healthy tissues. This review therefore maps the physiological networks that maintain amino acid balance, examines their disruption across disease states, and evaluates how these insights may guide precision metabolic therapies.

## 2 Biological Basis and Regulatory Pathways of Amino Acid Metabolism

This section defines the framework used throughout the review. We classify amino acids by nutritional requirement and metabolic fate, then connect anabolic and catabolic routes to rate‐limiting enzymes, nutrient‐sensing nodes, nitrogen disposal, and tissue allocation. The discussion moves from chemical identity to pathway flux and interorgan exchange, so that later disease sections can distinguish primary pathway defects from secondary shifts in circulating amino acid profiles.

### 2.1 Classification and Physiological Roles of Amino Acids

Amino acids function as the primary structural and metabolic units of mammalian systems, with their classification grounded in nutritional requirements and metabolic outcomes. Nutritional frameworks categorize these molecules into three distinct subsets based on endogenous biosynthetic capacity. EAAs must be acquired via dietary intake, as human pathways are insufficient to meet physiological demands [32]. This group includes BCAAs, which serve as key regulators of muscle proteostasis. Nonessential amino acids (NEAAs) are synthesized endogenously in sufficient quantities under basal conditions, whereas conditionally EAAs become rate‐limiting during periods of rapid growth or severe physiological stress [33, 34, 35, 36]. Notably, Gln and glutamate (Glu) operate as central mediators of interorgan nitrogen flux and immune cell function.

**FIGURE 1:**
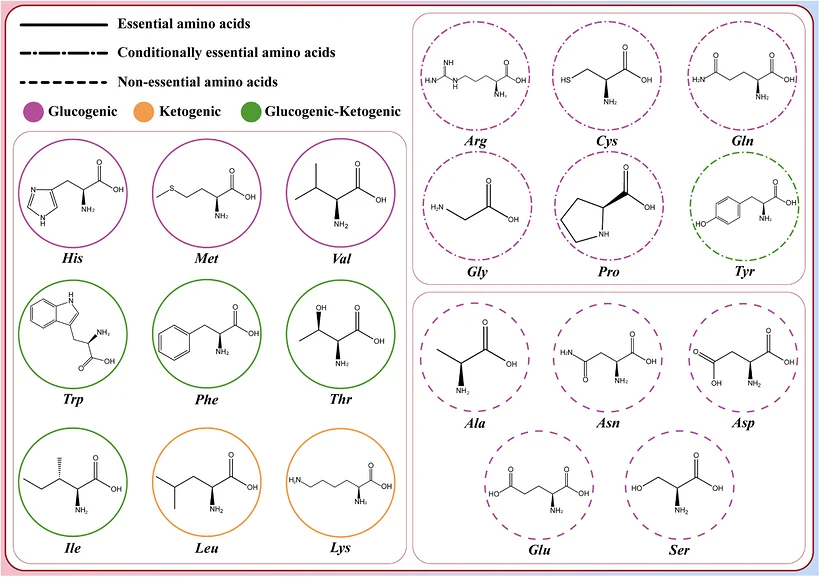
Classification of proteinogenic amino acids by nutritional requirement and metabolic fate. This figure classifies the 20 proteinogenic amino acids into essential, conditionally essential, and nonessential amino acids, and indicates their metabolic fate as glucogenic, ketogenic, or glucogenic–ketogenic. Solid, dash–dotted, and dashed outlines denote essential, conditionally essential, and nonessential amino acids, respectively; purple, orange, and green outlines denote glucogenic, ketogenic, and glucogenic–ketogenic amino acids, respectively. AA, amino acid; AAs, amino acids; Ala, alanine; Arg, arginine; Asn, asparagine; Asp, aspartate; Cys, cysteine; Gln, glutamine; Glu, glutamate; Gly, glycine; His, histidine; Ile, isoleucine; Leu, leucine; Lys, lysine; Met, methionine; Phe, phenylalanine; Pro, proline; Ser, serine; Thr, threonine; Trp, tryptophan; Tyr, tyrosine; Val, valine.

Further classification based on metabolic fate distinguishes amino acids by their degradation products and contribution to systemic energy balance. Glucogenic amino acids yield pyruvate or tricarboxylic acid (TCA) cycle intermediates to support gluconeogenesis [37, 38, 39]. Strictly ketogenic species, specifically leucine (Leu) and lysine (Lys), are catabolized into acetyl‐CoA or acetoacetate to generate ketone bodies [40]. A third subset, including aromatic amino acids and threonine, provides flexibility for energy production during fasting or metabolic stress [41, 42]. These complementary classifications are summarized in Figure 1.

Beyond their structural requirement for protein synthesis, amino acids coordinate systemic homeostasis through two primary mechanisms. First, they provide substrates for oxidative phosphorylation (OXPHOS) and the TCA cycle across various tissues. Second, they serve as precursors for bioactive compounds, including nucleotides, neurotransmitters, and hormones [43, 44]. Specific metabolic fluxes, such as Met‐derived methyl donors and cysteine (Cys)‐dependent GSH synthesis, integrate amino acid availability with epigenetic regulation and redox stability. Current research highlights BCAAs, sulfur‐containing amino acids, and the Gln–Trp axis as critical signaling nodes in immune modulation and cell fate determination [45].

### 2.2 Anabolic and Catabolic Pathways

Anabolism primarily drives de novo NEAA synthesis from central carbon metabolites [46]. Reversible transamination yields alanine (Ala), aspartate (Asp), and Glu directly from glycolysis and TCA cycle intermediates [47]. Gln synthetase subsequently converts Glu into Gln, supporting a primary systemic nitrogen carrier [48]. Alternatively, Ser biosynthesis branches from the glycolytic intermediate 3‐phosphoglycerate via phosphoglycerate dehydrogenase—a critical node frequently hijacked in pathogenesis [49]. Additional pathways derive proline (Pro) and Arg from Glu and citrulline, or Cys via Met–Ser integration [50]. Beyond maintaining substrate pools, these synthetic routes shape cellular redox capacity and epigenetic landscapes during stress.

Amino acid catabolism operates via three primary mechanisms. Aminotransferase‐mediated transamination redistributes nitrogen, removing amino groups to yield carbon skeletons for immediate energy utilization [51, 52]. Subsequent oxidative deamination, largely governed by Glu dehydrogenase, releases free ammonia while generating α‐KG to fuel the TCA cycle [53, 54]. Parallel decarboxylation pathways generate bioactive amines—such as histamine, dopamine, or γ‐aminobutyric acid (GABA)—integrating systemic metabolic flux with neuroendocrine signaling [55]. Collectively, these degradative steps provide intermediates for gluconeogenesis, lipogenesis, and cellular respiration.

The continuous release of cytotoxic ammonia during catabolism necessitates rapid hepatic detoxification [56]. The urea cycle executes this clearance via five sequential enzymatic steps, including carbamoyl phosphate synthetase 1 and arginase 1 [57]. By condensing free nitrogen and bicarbonate into excretable urea, this liver‐specific pathway preserves systemic nitrogen tolerance. Enzymatic deficiencies here precipitate hyperammonemia and severe metabolic toxicity, highlighting this cycle as a fundamental pathogenic vulnerability [58, 59, 60].

One‐carbon metabolism integrates specific amino acid fluxes to drive tetrahydrofolate‐dependent methylation [61]. By supplying methyl and formyl groups, this network fuels nucleotide biosynthesis and provides substrates for DNA and histone modifications [62]. Rate‐limiting enzymes like Ser hydroxymethyltransferase and methylenetetrahydrofolate reductase govern this biochemical relay. Thus, one‐carbon flux directly couples nutrient availability to genomic stability and epigenetic programming, acting as a central determinant of cellular transformation [63, 64].

### 2.3 Enzymes and Regulatory Nodes

Specific rate‐limiting enzymes govern metabolic flux across tissues to maintain systemic coordination. Transaminases mediate reversible nitrogen redistribution, whereas Glu dehydrogenase couples oxidative deamination to TCA cycle anaplerosis and ammonia release [65, 66]. Extrahepatic tissues rely on the branched‐chain α‐keto acid (BCKA) dehydrogenase complex to restrict BCAA degradation [45, 67]. Arg partitioning is tightly controlled by distinct arginase isoforms, with hepatic arginase 1 driving urea synthesis and extrarenal arginase 2 modulating inflammatory signaling [68, 69]. In addition, sulfur metabolism is continuously managed by cystathionine β‐synthase and γ‐lyase [70]. In the context of de novo synthesis, phosphoglycerate dehydrogenase diverts glycolytic carbon toward Ser production, representing a metabolic vulnerability frequently exploited by rapidly proliferating cells [71]. Additional key mediators include glutaminase (GLS) for Gln‐to‐Glu conversion and indoleamine 2,3‐dioxygenase 1 (IDO1), which commits Trp to the kynurenine (Kyn) pathway [72, 73].

Metabolic flux adapts dynamically through highly integrated regulatory layers. Substrate availability provides immediate feedback control over enzymatic activity, ensuring that metabolic rates match dietary and intracellular nutrient states [74]. Systemically, hormones orchestrate tissue‐level responses; insulin stimulates cellular uptake and protein synthesis, while fasting triggers glucagon and glucocorticoids to mobilize catabolism and ureagenesis [75]. At the molecular level, nutrient‐sensing networks involving activating transcription factor 4, general control nonderepressible 2 (GCN2), mTORC1, and AMP‐activated protein kinase (AMPK) reprogram transcription to align gene expression with physiological demand [76, 77]. At the same time, local metabolites enable rapid, transcription‐independent allosteric tuning [78].

Together, these mechanisms stabilize amino acid flux across changing nutritional and hormonal states. Genetic, transcriptional, or endocrine disruption can create substrate imbalance and impair nitrogen disposal. By reshaping bioenergetics, redox tone, and epigenetic state, enzyme dysregulation can contribute to disease and expose candidate diagnostic or therapeutic nodes.

### 2.4 Tissue Distribution and Function

Functioning as the central regulatory hub, the liver mediates the urea cycle to enable efficient ammonia detoxification [56, 79]. Hepatic pathways govern the de novo synthesis of all NEAAs and orchestrate gluconeogenesis from glucogenic substrates during fasting [80]. By dynamically storing and releasing substrates in response to physiological demand, hepatic metabolism effectively buffers plasma amino acid fluctuations [81].

Skeletal muscle is the main site for the breakdown and metabolism of BCAAs. This capacity stems from the strong local expression of specific aminotransferases and the BCKA dehydrogenase complex [82]. Beyond local degradation, muscle tissue acts as a major systemic nitrogen reservoir. During metabolic stress, it mobilizes Ala and Gln to fuel hepatic gluconeogenesis and support peripheral energy requirements [39, 83].

The kidneys maintain circulating pools by reabsorbing nearly all filtered amino acids [84]. Beyond reabsorption, renal Gln catabolism actively regulates systemic acid–base balance. The localized deamination of Gln generates ammonia that buffers tubular organic acids and stabilizes systemic pH [85]. This renal adaptation becomes particularly crucial during metabolic acidosis or chronic kidney dysfunction, conditions that profoundly remodel amino acid reabsorption and flux [86, 87].

The intestinal tract serves as the initial site of dietary assimilation and a critical interface with the gut microbiota. Epithelial cells absorb luminal substrates and directly utilize a substantial fraction to sustain continuous mucosal renewal [88]. At the same time, microbial fermentation pathways generate bioactive metabolites that compete or synergize with host absorption mechanisms. This bidirectional host–microbiota crosstalk fundamentally shapes systemic amino acid availability [89, 90].

Neural tissues require selective amino acid transport across the blood–brain barrier to sustain localized metabolic demands [91]. Within the central nervous system, amino acids function as obligate neurotransmitter precursors. Glu and glycine (Gly) directly mediate synaptic transmission, whereas tyrosine (Tyr) and Trp facilitate the synthesis of catecholamines and serotonin [92, 93, 94]. Tight regulation of these intracellular pools ensures neuronal survival and redox stability despite limited local biosynthetic capacity [95, 96].

**FIGURE 2:**
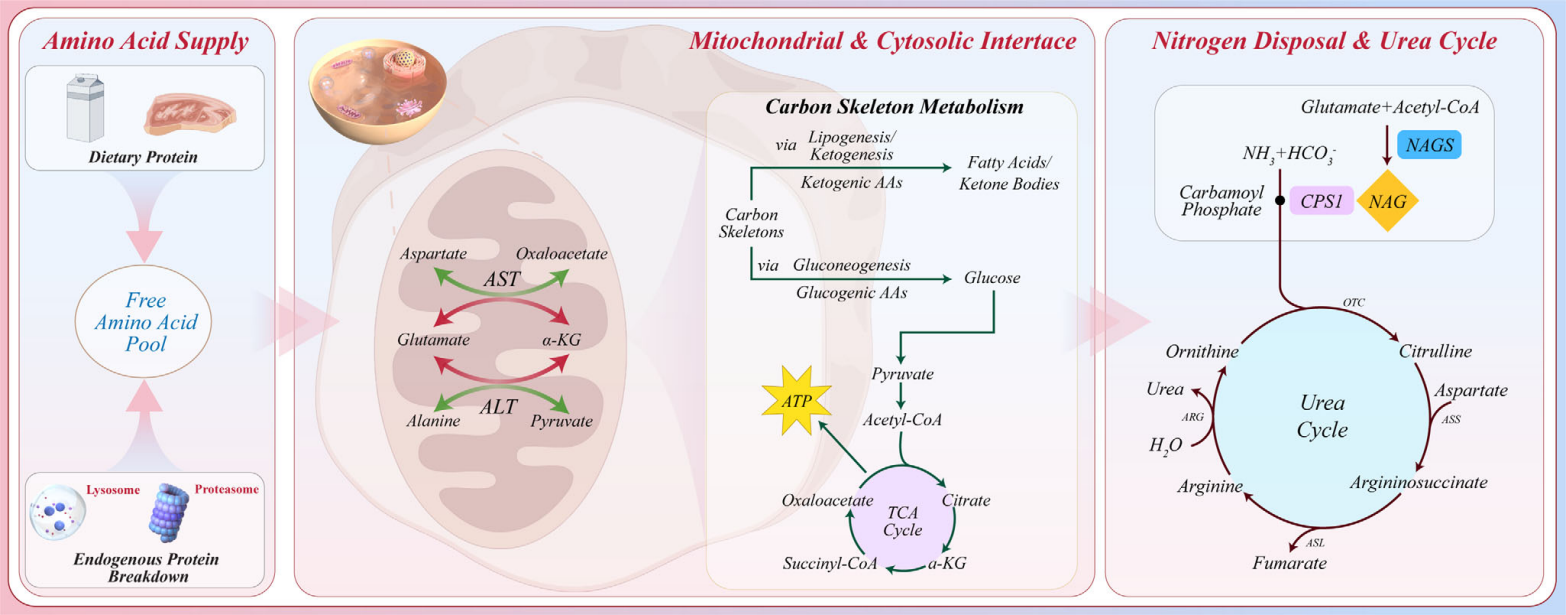
Core pathways maintaining mammalian amino acid homeostasis. This figure summarizes the major sources and metabolic routes that sustain systemic amino acid homeostasis, including dietary protein intake, endogenous protein breakdown, the free amino acid pool, mitochondrial and cytosolic transamination, carbon skeleton metabolism, and nitrogen disposal through the hepatic urea cycle. AA, amino acid; AAs, amino acids; Acetyl‐CoA, acetyl‐coenzyme A; ALT, alanine aminotransferase; ARG, arginase; ASL, argininosuccinate lyase; ASS, argininosuccinate synthase; AST, aspartate aminotransferase; ATP, adenosine triphosphate; α‐KG, α‐ketoglutarate; CPS1, carbamoyl phosphate synthetase 1; HCO^3−^, bicarbonate; NAG, N‐acetylglutamate; NAGS, N‐acetylglutamate synthase; NH_3_, ammonia; OTC, ornithine transcarbamylase; TCA, tricarboxylic acid.

These anatomical compartments form an interdependent metabolic network. Circulating substrates and endocrine signals allow each organ to buffer local demand while contributing to whole‐body amino acid balance. Disruption of local enzyme activity or interorgan crosstalk can therefore propagate beyond the affected tissue and contribute to systemic metabolic disease (Figure 2).

Amino acid metabolism is therefore better conceptualized as an allocation system rather than a set of isolated reactions. The most robust mechanistic links involve nitrogen handling, BCAA catabolism, one‐carbon flux, redox buffering, and transporter‐mediated organ exchange. Causality remains difficult to assign, because circulating amino acid levels may reflect altered intake, tissue release, microbial metabolism, or impaired clearance. Future work should integrate isotope tracing, tissue‐resolved metabolomics, and genetic perturbation to distinguish whether each observed change is a driver, compensation, or biomarker.

## 3 Amino Acid Metabolism in Physiological Homeostasis

### 3.1 Nervous System

Amino acids function as required precursors for synaptic neurotransmitters to mediate circuit stability [97]. In presynaptic neurons, GLS converts Gln into Glu to drive primary excitatory transmission. Astrocytes continuously manage this excitatory flux through the astrocyte–neuron Glu–Gln cycle. These glial cells clear synaptic Glu and utilize Gln synthetase to regenerate Gln for neuronal delivery, thereby preventing excitotoxicity [98, 99]. Beyond direct excitation, Glu undergoes decarboxylation via Glu decarboxylase to yield GABA for principal inhibitory signaling [100]. At the same time, Ser‐derived Gly operates within the spinal cord and brainstem to synergize with GABA and maintain global neural excitability [101].

Aromatic amino acids supply carbon structures for monoamine neurotransmitters governing cognitive and behavioral states. Trp acts as the rate‐limiting substrate for serotonin synthesis via Trp hydroxylase. This dependency directly couples central serotonin levels to circulating dietary Trp, functionally linking nutritional intake with emotional and circadian regulation [102, 103]. At the same time, phenylalanine (Phe) hydroxylation yields Tyr to support catecholamine production. Tyr hydroxylase converts this substrate into L‐DOPA, which subsequently undergoes sequential metabolism to generate dopamine for motor and reward circuitry alongside norepinephrine for attention and stress responses [104, 105].

Developing neural networks rely on continuous amino acid transport for structural and functional maturation. Circulating BCAAs cross the blood–brain barrier to activate mechanistic target of rapamycin signaling, thereby promoting neurogenesis and synaptic plasticity while supplying structural components for membrane biogenesis [106, 107]. Sulfur‐containing amino acids execute parallel regulatory functions. Methionine‐derived methyl groups shape the epigenetic landscape during neuronal and oligodendrocyte differentiation. Simultaneously, Cys availability dictates the synthesis of GSH to shield developing neural populations from oxidative stress [108, 109].

Cerebral energy demands further necessitate local amino acid use. The Glu–Gln cycle intrinsically links neurotransmitter recycling with astroglial bioenergetics by providing substrates for TCA cycle and ATP production [110]. During periods of hypoglycemia or systemic fasting, ketogenic substances including Leu and Lys produce ketone bodies that enhance metabolism to maintain neuronal vitality [111].

Together, these pathways maintain neurochemical stability. Enzyme or transporter disruption can disturb excitation–inhibition balance, energy metabolism, and redox control, creating a shared metabolic basis for neurological disease.

### 3.2 Cardiovascular System

Endothelial homeostasis relies on targeted amino acid utilization to preserve circulatory stability. The conditionally EAA L‐Arg functions as the primary substrate for endothelial nitric oxide (NO) synthase. This enzyme catalyzes NO synthesis to regulate physiological vascular tone [112, 113]. Thus, Arg‐dependent NO generation dictates endothelium‐mediated vasodilation and preserves barrier integrity. This pathway further limits vascular smooth muscle proliferation while restricting platelet adhesion, thereby preventing pathological remodeling and sustaining vessel patency [114]. At the same time, homocysteine (Hcy) acts as a functional intermediate in sulfur‐containing metabolism. At physiological concentrations, it mitigates oxidative stress and facilitates structural collagen cross‐linking. This metabolic intermediate also supplies methyl groups to shape dynamic endothelial transcription profiles [115].

Continuous myocardial contraction demands sustained energetic support from amino acids. Cardiomyocytes actively metabolize BCAAs through dedicated enzymatic pathways to yield carbon skeletons for TCA cycle. This continuous flux supplements ATP generation under both basal conditions and elevated hemodynamic loads [116, 117]. These branched‐chain substrates additionally optimize excitation‐contraction coupling by regulating intracellular calcium transients. They simultaneously activate mechanistic target of rapamycin signaling to dictate structural protein turnover [118]. Beyond branched‐chain species, localized Glu and Asp drive the malate–Asp shuttle to transfer cytoplasmic reducing equivalents into mitochondria for sustained OXPHOS [119]. Direct Glu oxidation further fuels the TCA cycle to support steady‐state energy production for uninterrupted contractile output [120].

Circulatory stability further necessitates metabolic control over coagulation. Hepatic pathways utilize systemic amino acids to synthesize circulating procoagulant and anticoagulant proteins [121]. Specifically, the biological activity of multiple coagulation factors depends on the vitamin K‐dependent γ‐carboxylation of localized glutamic acid residues. This essential post‐translational modification permits structural calcium binding to enable physiological clot formation [122]. In the peripheral circulation, endothelial Arg flux generates NO to modulate platelet activation dynamically. Parallel utilization of Gly and Ser supplies structural precursors for platelet membrane phospholipids. Together, these localized metabolic actions optimize cellular function to avert spontaneous thrombosis [123, 124].

In the cardiovascular system, amino acid metabolism links endothelial signaling, myocardial energetics, and coagulation control. Disturbance of these linked processes can impair vascular tone, contractile performance, and thrombotic balance, providing mechanistic entry points for cardiovascular disease.

### 3.3 Digestive System

Mucosal barrier function relies on continuous amino acid utilization to preserve structural defenses. Gln serves as the primary bioenergetic substrate for rapidly renewing intestinal epithelium. GLS‐mediated mitochondrial oxidation of this substrate fulfills the majority of enterocyte energy requirements [125]. This metabolism drives epithelial proliferation while concurrently upregulating the transcription of specific tight junction proteins to reinforce mechanical barrier integrity. Thus, this localized Gln utilization actively prevents the paracellular translocation of luminal antigens [126]. Arg metabolism further supports mucosal homeostasis. NO synthase utilizes Arg to generate physiological NO for regional blood flow regulation and epithelial repair. At the same time, Arg functions as a required precursor for polyamine synthesis to mediate enterocyte renewal [127, 128]. Together, these pathways secure the structural integrity necessary for subsequent digestive and absorptive processes.

Pancreatic acinar cells require amino acid‐supported energy metabolism to sustain enzyme synthesis and secretion [129]. In isolated rat pancreatic acini, selected amino acids can serve as safe energy substrates, supporting acinar bioenergetics without implying that all amino acid classes directly maintain luminal enzyme output [130]. Within the hepato‐biliary compartment, portal‐derived amino acids regulate bile acid dynamics. Cys provides the essential precursor for taurine (Tau) synthesis to facilitate bile acid conjugation. This modification promotes micelle formation and intestinal lipid absorption. Additional hepatic amino acid utilization supports binding proteins and membrane transporters that maintain enterohepatic circulation and physiological biliary excretion [131, 132].

The host–microbiota connection is mediated by integrated amino acid metabolism. Commensal bacteria use unabsorbed dietary and host‐derived nitrogenous substrates to generate metabolites with homeostatic effects [133]. Microbial Trp metabolism yields indole derivatives that act as endogenous aryl hydrocarbon receptor (AhR) ligands and support mucosal repair and immune tolerance [134, 135]. In mammalian systems, urea–nitrogen salvage and microbial EAA provisioning are best viewed as context‐dependent nutrient‐recycling mechanisms rather than universal additions to the host amino acid pool [136, 137]. Community composition, host physiology and diet therefore determine whether microbial biosynthesis measurably contributes to systemic EAA availability.

In the digestive system, amino acids support epithelial renewal, pancreatic enzyme production, bile acid conjugation, and host–microbiota exchange. Failure of this network can weaken barrier function, impair absorption, and amplify inflammatory or metabolic disease.

### 3.4 Metabolic and Endocrine System

Metabolic pathways continuously govern systemic carbohydrate and lipid homeostasis across fed and fasted states. BCAAs maintain physiological insulin sensitivity within specific insulin‐responsive tissues. These substrates modulate insulin receptor cascade activity while preserving glucose transporter membrane localization across skeletal muscle and adipose depots [138]. During periods of fasting, Ala operates as the primary circulating substrate for hepatic gluconeogenesis. This hepatic utilization ensures euglycemia and averts hypoglycemic events [39]. Gln contributes to glycemic control by sustaining pancreatic beta‐cell function and regulating hepatic glucose output. This molecule simultaneously provides substrates for the TCA cycle across corresponding target organs [139]. Within adipose depots, these pathways regulate distinct aspects of lipid handling. They mediate de novo lipogenesis and lipolysis while orchestrating thermogenic activation [140]. Branched‐chain substrates propel the differentiation and metabolic activation of brown adipose tissue to support adaptive thermogenesis and systemic energy expenditure. At the same time, Ser and Gly supply required precursors for adipocyte membrane biosynthesis. Gln additionally modulates lipolytic rates to maintain circulating fatty acids within physiological ranges [141, 142].

Amino acids act as essential precursors for endocrine hormone biosynthesis. Their availability shapes hormonal output and systemic signaling. Tyr provides the required precursor for thyroid hormone synthesis. These hormones subsequently govern systemic metabolic rates, guide developmental programming, and dictate energy expenditure [143]. Tyr additionally supplies the substrate for adrenal catecholamine production. The resulting epinephrine and norepinephrine mediate physiological stress responses and coordinate adaptive metabolic shifts [144]. Trp operates as the essential precursor for pineal melatonin synthesis. This hormone coordinates circadian rhythms, dictates sleep–wake cycles, and preserves associated metabolic stability [145, 146]. In addition, Arg modulates the secretion of specific pituitary hormones, including growth hormone and gonadotropin‐releasing hormone. These signaling molecules ensure physiological growth, preserve reproductive function, and stimulate systemic anabolism [147]. These substrate‐dependent pathways create an explicit mechanistic link between dietary nutrient availability and systemic endocrine regulation.

Substrate availability critically sustains physiological pancreatic islet function and insulin dynamics. The entire spectrum of amino acids provides the structural components for preproinsulin synthesis within beta cells. Adequate substrate supply remains a strict prerequisite for maintaining physiological insulin stores [148]. Specific molecules, including BCAAs and Arg, augment glucose‐stimulated insulin secretion under physiological conditions. These substrates operate simultaneously as nutrient signals and energetic fuels to facilitate insulin exocytosis [149, 150]. Gln sustains beta‐cell redox stability and overall cellular survival. This local metabolism ensures the functional beta‐cell mass required for glycemic control. These synergistic effects establish a feedforward loop coupling nutrient availability to insulin secretion. This mechanism supports the coordinated handling of all major macronutrients following dietary intake [151].

Amino acid use in metabolic and endocrine tissues integrates nutrient availability with glucose handling, lipid turnover, hormone production, and insulin secretion. When this coordination fails, amino acid imbalance can worsen endocrine dysfunction and metabolic disease.

### 3.5 Immune System

Innate and adaptive immunity rely on amino acids to sustain bioenergetic requirements and biosynthetic output. Gln operates as the primary oxidative substrate for rapidly dividing immune populations. This utilization sustains T‐cell clonal expansion and facilitates proinflammatory cytokine generation. Parallel Gln metabolism within macrophages supports robust phagocytosis and antimicrobial mediator secretion [152, 153]. Arg additionally shapes adaptive responses by modulating T‐cell receptor signaling cascades to promote CD4+ and CD8+ T‐cell proliferation. Arg utilization preserves the bactericidal capacity of tissue‐resident macrophages to sustain innate cellular defense [154, 155]. In addition, BCAAs supply required carbon and nitrogen scaffolds for de novo macromolecule biosynthesis. These substrates propel B cell antibody production and affinity maturation, while augmenting the cytotoxic output of natural killer cells [156].

Substrate use continuously shapes the critical balance between immune activation and physiological tolerance. Trp degradation via the IDO1‐dependent Kyn pathway explicitly modulates the ratio of effector to regulatory T cells. This catabolic route constrains excessive initial responses and facilitates tissue resolution following pathogen clearance [157]. At the same time, sulfur‐containing amino acids supply required precursors for intracellular antioxidant defense. Methionine and Cys specifically regulate GSH biosynthesis to preserve redox stability [158]. This localized redox control protects antigen‐presenting cells from oxidative dysfunction to ensure sustained host defense during severe infection [159, 160].

Immune homeostasis depends on a narrow balance between amino acid‐supported activation and metabolic restraint. Perturbing this balance can shift cells toward excessive inflammation, impaired tolerance, or defective host defense.

### 3.6 Skeletal System

Proteinogenic substrates serve as the primary building blocks for bone matrix generation. Within this organic scaffold, Type I collagen requires an extraordinary abundance of Gly to achieve stable triple helix assembly [161, 162]. Pro and Lys subsequently undergo targeted post‐translational hydroxylation to enable critical intermolecular fiber cross‐linking. This essential structural modification establishes the required architecture for subsequent hydroxyapatite crystal deposition and proper matrix mineralization [163]. Through these interconnected synthetic steps, substrate use supports the physical resilience necessary to withstand physiological mechanical loading.

Beyond providing structural scaffolds, targeted substrate catabolism orchestrates the functional interplay between bone‐forming and bone‐resorbing populations. Proliferating osteoblasts rely heavily on Gln oxidation to generate sufficient adenosine triphosphate for energy‐intensive matrix synthesis [164]. This same molecule supplies required nitrogenous precursors to support nucleotide and glycosaminoglycan biosynthesis during cellular expansion [165]. In parallel, Arg metabolism yields physiological NO via endothelial NO synthase activity. The resulting signaling cascades enhance osteoblastic mineralization capacity while actively suppressing excessive osteoclast activation [166]. This dual regulatory mechanism maintains the balanced tissue turnover necessary for structural repair.

Circulating amino acids further dictate bone mineralization by modulating systemic calcium and phosphorus metabolism. Endocrine regulation of these minerals depends upon the continuous synthesis of parathyroid hormone from available proteinogenic precursors [167]. Parallel substrate utilization influences renal 1α‐hydroxylase activity to control vitamin D activation and subsequent intestinal mineral absorption. This intricate endocrine coordination supports the precise systemic mineral microenvironment required for proper hydroxyapatite deposition [168, 169].

The skeletal system thus relies on a highly integrated metabolic network to synthesize structural matrices and govern localized cellular interactions. Maintaining these pathways ensures appropriate systemic mineral balance alongside lifelong mechanical integrity. Disrupting substrate use severely uncouples osteogenic activity from osteoclast‐mediated resorption. Such physiological imbalances ultimately compromise matrix architecture to precipitate the onset of severe bone pathophysiology.

### 3.7 Urinary System

Continuous tubular reabsorption establishes the primary mechanism for maintaining organism‐wide amino acid availability. Proximal tubular epithelia reclaim virtually all filtered substrates through specific apically localized sodium‐dependent and sodium‐independent transport mechanisms [170]. These diverse transport networks selectively recognize neutral, acidic, and basic molecular structures to orchestrate precise substrate retrieval. Such selective reclamation precisely adjusts circulating concentrations to align with fluctuating nutritional supply and physiological energy demands [171, 172, 173]. By preventing excessive urinary substrate loss, this active reabsorption safeguards the precursor pools required for strong protein synthesis and complex biochemical reactions throughout the organism.

Beyond substrate reclamation, localized renal catabolism operates as the primary physiological regulator of systemic acid–base equilibrium. Within this regulatory network, Gln degradation serves as the effector mechanism. Proximal tubular cells actively internalize both filtered and circulating Gln to undergo continuous mitochondrial oxidation. Sequential processing via GLS and Glu dehydrogenase yields significant quantities of free ammonia [174]. This ammonia subsequently diffuses into the tubular lumen to combine with free hydrogen ions for immediate urinary excretion. At the same time, these oxidative steps generate equimolar bicarbonate ions for continuous transport into the systemic circulation. This newly synthesized bicarbonate acts to buffer continuous endogenous acid production [175]. Renal sensing mechanisms dynamically adjust this Gln utilization in response to subtle systemic pH variations to ensure tight homeostatic control under varying physiological conditions.

High‐capacity solute transport demands extraordinary metabolic activity from tubular epithelial populations. To meet this intense bioenergetic requirement, intrinsic cellular networks rely heavily on continuous Gln oxidation for localized energy generation [176]. Parallel substrate utilization involves sulfur‐containing amino acids and Gly as required precursors for GSH biosynthesis. This intracellular antioxidant network actively preserves tubular redox stability to protect delicate epithelial structures from continuous oxidative stress [177]. Together, these interconnected bioenergetic and protective metabolic routes ensure sustained tubular survival and strong reabsorptive capacity.

The mammalian kidney thus operates as a highly integrated metabolic hub combining systemic physiological regulation with intrinsic structural maintenance. Continuous coordination between tubular reabsorption and localized catabolism supports organism‐wide substrate availability while preserving acid–base equilibrium. Thus, enzymatic or transport failures severely impair systemic homeostasis and compromise intrinsic renal survival. Such profound physiological disruptions actively precipitate the onset and progression of profound nephrological and systemic pathologies.

### 3.8 Reproductive System

Within the male reproductive tract, Arg utilization supports sperm motility, capacitation, and acrosome reaction dynamics, thereby contributing to successful fertilization [178, 179]. In the ovary, mouse and human multiomics studies link oocyte competence and aging to translational, mitochondrial, and amino acid‐related metabolic states [179]. Pro serves as a structural component for sperm nuclear protamine assembly. These proteins mediate chromatin compaction and preserve genomic integrity during spermatogenesis [180]. Following fertilization, BCAAs regulate cell‐cycle transitions within preimplantation embryos and help support blastocyst formation. By shaping biosynthetic capacity, these pathways contribute to embryonic cell fate specification [181].

Beyond gamete maturation, targeted substrate catabolism governs the biogenesis of endocrine signals necessary for coordinating the hypothalamic–pituitary–gonadal axis. Ser and Gly supply required methyl donors and isoprenoid intermediates through one‐carbon metabolic routes. These biochemical contributions promote de novo cholesterol synthesis. This newly synthesized cholesterol serves as the required precursor for subsequent estrogen and progesterone production alongside continuous testosterone generation [182, 183]. Tyr provides the core precursor for pituitary gonadotropin synthesis. These circulating endocrine signals subsequently propel gonadal steroidogenesis to coordinate ongoing gamete maturation. Parallel Tyr utilization facilitates the generation of distinct peptide hormones required for rhythmic reproductive cycle regulation [184].

During pregnancy, amino acid metabolism and transport support placental development and sustained nutrient delivery to the fetus. Trophoblast populations express specialized transport systems to mediate the directional transfer of proteinogenic substrates from the maternal circulation. This active transport continuously aligns nutritional supply with escalating fetal growth demands [185, 186]. Arg and Gln specifically facilitate physiological trophoblast invasion. This localized utilization simultaneously promotes subsequent placental vascular remodeling. Such structural adaptations ensure adequate uteroplacental perfusion to optimize ongoing nutrient exchange [187]. In addition, circulating substrates provide the required scaffolds for gestational peptide hormone biosynthesis. These endocrine signals maintain corpus luteum function to safeguard the progression of normal pregnancy [188, 189].

**FIGURE 3:**
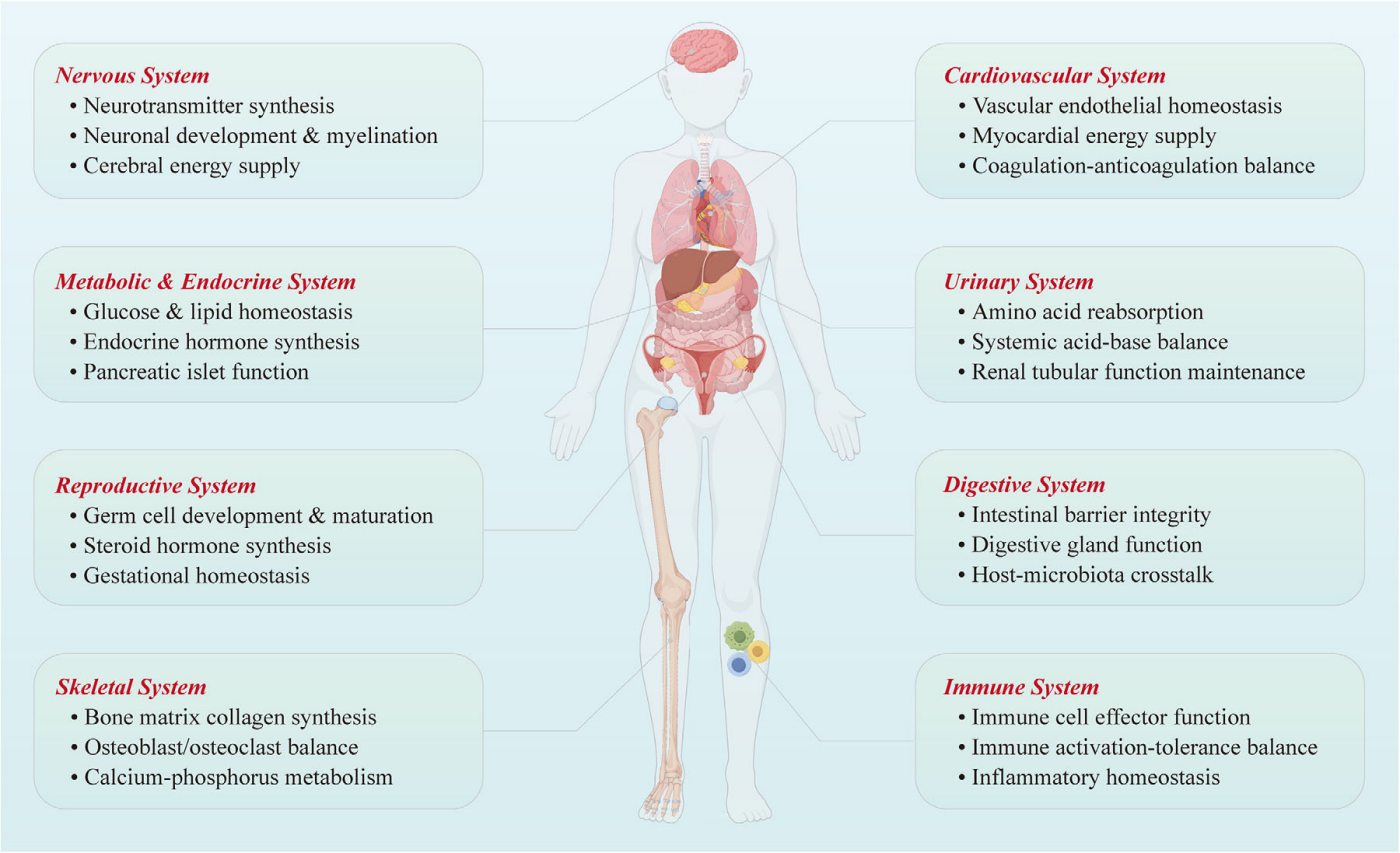
Amino acid metabolism in the maintenance of physiological homeostasis across major mammalian organ systems. This figure summarizes the core physiological functions of amino acid metabolism in maintaining homeostasis across eight major mammalian organ systems, including the nervous, cardiovascular, digestive, metabolic and endocrine, immune, skeletal, urinary, and reproductive systems, outlining the essential role of amino acid metabolic pathways in sustaining organ‐specific physiological activities.

Integrated substrate utilization continuously orchestrates these interconnected physiological events to sustain lifelong mammalian reproductive competence. Precise metabolic tuning within localized tissues ensures the coordinated function of the entire gonadal axis. This regulation actively preserves gamete development alongside strong gestational support systems. Thus, enzymatic dysregulation severely disrupts these tightly controlled physiological processes. Such metabolic failures actively compromise overall reproductive capacity to precipitate profound pathophysiological conditions (Figure 3).

Across organ systems, amino acids serve three recurring physiological roles: substrate supply, signal transduction, and stress buffering. However, the evidence is uneven across tissues. Neural, immune, renal, and metabolic–endocrine systems have relatively clear mechanistic links, whereas reproductive and skeletal contexts still rely more heavily on animal or associative studies. A central unresolved question is how local amino acid pools are prioritized when systemic demand changes during fasting, inflammation, pregnancy, or aging.

## 4 Mechanisms Underlying Amino Acid Metabolic Homeostasis

### 4.1 Energy and Redox Homeostasis

Under conditions of energy deficiency, nutritional stress and oxidative stress, amino acid metabolism serves as a regulatory hub for cellular energy homeostasis, redox balance, and survival. When energy supply is impaired or glycolysis is inhibited, Gln is converted to Glu and α‐KG through GLS and Glu dehydrogenase, replenishing the TCA cycle when glucose‐derived ATP production is limited [190, 191, 192]. In mammalian cancer‐cell models, impaired respiratory metabolism can increase reliance on Gln‐dependent TCA cycle flux, supporting the broader principle that Gln catabolism helps maintain OXPHOS under metabolic stress [192]. BCAAs are additional carbon sources for TCA cycle anaplerosis under stress. BCAAs are catabolized by branched‐chain aminotransferase (BCAT) and the BCKA dehydrogenase complex to generate acetyl‐CoA and succinyl‐CoA, which enter the TCA cycle in energy‐demanding tissues including myocardium and skeletal muscle [82, 193]. Under stress conditions, the BCKDK inhibitor BT2 relieves phosphorylation‐mediated inhibition of branched‐chain α‐keto acid dehydrogenase (BCKDH) complex, enhances BCAA oxidation and improves energy homeostasis in heart and kidney [194, 195]. Amino acids including Ala and Asp can also replenish TCA cycle intermediates via transamination reactions during cellular energy stress.

Amino acid metabolism is essential for cellular redox homeostasis and oxidative‐stress control. In mammalian cell and cardiac models, Gln‐ and BCAA‐linked metabolic flux shapes mitochondrial function, inflammatory signaling and reactive oxygen species (ROS) handling. Gln‐derived α‐KG and itaconate suppress inflammatory ROS production and pyroptosis, linking anaplerotic metabolism to antioxidant defense [196]. Conversely, accumulation of BCAAs and their α‐keto acid derivatives exacerbates mitochondrial oxidative stress and lipid peroxidation, increasing susceptibility to myocardial ischemia/reperfusion injury. Activation of BCAA catabolism reduces ROS production and improves myocardial prognosis [195, 197].

The mTORC1 integrates amino acid availability, cellular energy status and redox homeostasis. The mTORC1 senses intracellular amino acid levels via the Rag small GTPases, GATOR1/DEPDC5 and other regulatory complexes [198]. Genetic deletion of DEPDC5 leads to sustained mTORC1 activation even under stress conditions, which promotes mitochondrial respiration and cellular hypertrophy, indicating that the core function of mTORC1 is to drive anabolic metabolism [199]. BCAAs, especially Leu, activate mTORC1 via sensors including Sestrin2, LeuRS, and Rab1A. mTORC1 regulates insulin transcription in pancreatic β‐cells, as well as metabolic and inflammatory responses in cardiomyocytes and retinal cells [200, 201]. Amino acid imbalance induced by the antibiotic halofuginone triggers two parallel signaling cascades: it activates the GCN2‐mediated integrated stress response on the one hand, and sustains mTORC1 activation via elevated free amino acid levels on the other. Genetic deletion of GCN2 leads to the uncoupling of mTORC1 from the real nutritional and energetic status of cells, resulting in hepatic steatosis and cell death. This finding validates the core mechanism that cells upregulate amino acid transporters and catabolic enzymes, and activate stress response pathways to exert cytoprotective effects under nutrient restriction [202].

Cells undergo adaptive amino acid metabolic reprogramming to maintain survival under nutrient stress and deprivation. In skeletal muscle after exercise, the sensitivity of mTORC1 to Leu is sustained for at least 48 h, accompanied by the upregulation of Leu transporters and leucyl‐tRNA synthetase, which are core components of the amino acid sensing machinery. This finding indicates that transient nutrient stress primes cells to a long‐term state of hypersensitivity to amino acids, enabling rapid initiation of anabolic metabolism upon nutrient refeeding [200]. In multiple stress models including heat stress, copper nanotoxicity, and high‐fat load in the myocardium, downregulated glycolysis is consistently accompanied by the upregulation of amino acid transporters, as well as Gln and BCAA catabolic pathways, which coordinately support TCA cycle flux and antioxidant defense [203]. Notably, long‐term Gln deprivation paradoxically activates the Akt–mTOR pathway and inhibits autophagy, which accelerates cellular senescence, while mTOR inhibition reverses this effect. This finding further elucidates the core regulatory axis of nutrient stress–mTOR regulation–autophagy/cell survival in the adaptive response to nutrient deprivation [204].

These findings indicate that under energy deprivation, amino acids, especially Gln and BCAAs, replenish the TCA cycle to maintain mitochondrial OXPHOS and ATP production. Meanwhile, metabolites derived from sulfur‐containing amino acids and Gln support antioxidant defense to counteract oxidative stress and maintain cellular redox homeostasis. The mTORC1 drives synthetic metabolism and cell cycle processes in nutrient‐rich conditions, while under nutritional and energy stress, it coordinates with GCN2, autophagy, and other pathways to reprogram amino acid transport, catabolism, and antioxidant gene expression, ultimately maximizing cell survival and functional maintenance under adverse conditions.

### 4.2 Epigenetic Regulation

Amino acids are not only building blocks of proteins, but also upstream regulatory factors of epigenetics. By providing methyl, acetyl, and other chemical groups for epigenetic modifications and regulating DNA, histone, and RNA epigenetic markers through their bioactive metabolites and metabolic pathways, amino acids can establish long‐term gene‐expression programs.

S‐adenosyl methionine (SAM), the universal methyl donor generated from Met via one‐carbon metabolism, acts as a metabolic switch for cellular methylation capacity. In aged muscle stem cells, SAM depletion reduces repressive histone marks H3K9me2/3 and heterochromatin protein, causing heterochromatin loss, DNA damage accumulation, and impaired regeneration; these changes can be reversed by SAM supplementation or polyamine synthesis inhibition [205]. Ethionine‐induced SAM deficiency lowers global H3K27me3, activates histone demethylase UTX, inhibits β‐catenin signaling and impairs neural stem cell differentiation, leading to neural tube defects that are partly mitigated by SAM supplementation [206]. Dietary methyl donors exert long‐term epigenetic programming effects in mammalian systems: gestational folate and choline imbalance alters offspring hypothalamic SAM:S‐adenosyl homocysteine (SAH) ratio and global DNA methylation, whereas betaine supplementation in a multiple sclerosis model normalizes the SAM:SAH ratio, increases H3K4me3 and improves mitochondrial function [207, 208]. Together, these findings support a conserved link between Met availability, methyl‐donor balance and epigenetic regulation across metabolically active tissues [209].

Acetyl‐CoA, the sole acetyl donor for histone acetylation, is mainly derived from amino acid catabolism, directly linking amino acid availability to epigenetic gene regulation. In white adipose tissue of obese mice, reduced Gln and Met cause insufficient acetyl‐CoA and SAM, lowering H3K27ac and H3K4me3 at the BMAL1 promoter and suppressing circadian clock gene transcription, which is rescued by Gln or Met supplementation [210]. Individual EAAs reshape the histone acetylation landscape specifically. Dietary isoleucine (Ile) deficiency induces marked H2A/H4 hypoacetylation in mouse liver, with altered acetyl‐CoA metabolism and transcriptomic changes in chromatin and metabolic regulation, demonstrating that EAAs regulate metabolic gene expression via acetyl‐CoA‐mediated epigenetic programming in a highly specific manner [211].

Amino acid metabolic intermediates regulate epigenetic modifications, linking cellular metabolic status to chromatin state. Polyamine synthesis competes with methylation for SAM; excessive polyamine synthesis in muscle stem cells depletes SAM, causing heterochromatin loss and epigenetic aging, reversed by polyamine synthesis inhibition [205]. BCAA catabolism regulates histone demethylation via α‐KG, a key cofactor for histone demethylases. In the retina, BCAT1 activation consumes α‐KG, inhibits dioxygenase activity, enriches H3K4me3 at inflammatory gene promoters, and drives diabetic retinopathy inflammation, reversed by BCAT1 inhibition [212]. The nonproteinogenic amino acid α‐aminobutyric acid reprograms macrophage metabolism and elevates H3K27me3 at proinflammatory gene promoters, inhibiting M1‐type macrophage (M1) polarization and inflammatory responses [213].

Amino acid metabolism also regulates RNA epigenetic marks, especially N6‐methyladenosine (m6A), the most common internal mRNA modification in eukaryotes. In cancer, Met metabolism supplies SAM to drive m6A modification of PD‐L1 and V‐domain Ig suppressor of T‐cell activation mRNAs, promoting their translation and tumor immune evasion. Dietary Met restriction or m6A reader YTH N6‐methyladenosine RNA‐binding protein 1 inhibition reduces immune checkpoint expression, enhances CD8+ T‐cell infiltration, and synergizes with PD‐1 blockade for antitumor effects [214]. In aquatic animals, appropriate dietary Met modulates hepatic m6A via SAM, regulates autophagy and apoptosis gene expression, and maintains metabolic and antioxidant homeostasis [215].

These findings establish amino acid metabolism as a master upstream regulator of the cellular epigenetic landscape. The Met–one‐carbon axis, via modulating the SAM:SAH ratio, directly determines DNA, histone and RNA methylation status, with far‐reaching impacts on stem cell aging, embryonic development and disease pathogenesis. Meanwhile, catabolism of Gln, BCAAs and other amino acids generates key epigenetic regulators including acetyl‐CoA and α‐KG, which precisely control histone acetylation and demethylation to adapt to environmental changes.

### 4.3 Interdependence With Glucose and Lipid Metabolism

Amino acids including Ala, Gln, BCAAs, Ser, and Gly form a regulatory network with glucose and lipid metabolism and help maintain carbohydrate and lipid metabolic homeostasis. During fasting or overnutrition, these amino acids maintain blood glucose homeostasis through gluconeogenesis and the Ala glucose cycle, while providing carbon sources for fatty acid synthesis, ketone body production, and adipocyte differentiation. Amino acid, glucose, and lipid metabolism coordinate the overall energy homeostasis under different physiological and pathological conditions, and their dysregulation promotes the progression of various metabolic disorders.

Ala and Gln form a gluconeogenic network connecting skeletal muscle, liver, and kidney metabolism. During fasting or elevated gluconeogenic flux, skeletal muscle releases Ala and Gln, which are transported to the liver for glucose synthesis through the Cahill cycle and Gln‐driven gluconeogenesis [216]. Mice with annexin A6 deletion exhibit severe fasting and posthepatectomy hypoglycemia driven by impaired hepatic sodium‐coupled neutral amino acid transporter 4 transport and reduced hepatic Ala uptake, verifying the indispensable role of Ala‐dependent gluconeogenesis in stress‐induced glucose homeostasis maintenance [217, 218]. Pharmacological inhibition of Ala aminotransferase 2 markedly reduces hepatic Ala‐derived glucose production in diabetic murine models and alleviates hyperglycemia, identifying amino acid‐driven gluconeogenesis as an actionable therapeutic target for glycemic control [216]. Under conditions of blocked lactate and pyruvate transmembrane transport in basigin‐deficient mice, organisms compensate by increasing Ala and Gln utilization to replenish the TCA cycle, while upregulating fatty acid β‐oxidation and ketogenesis to ameliorate high‐fat diet‐induced hyperglycemia and hepatic steatosis. This adaptive substrate switching maintains systemic energy output under metabolic constraint [219]. Gln sustains renal and hepatic gluconeogenesis via conversion to α‐KG and subsequent TCA cycle anaplerosis across multiple stress models [220].

BCAAs are associated with bidirectional metabolism between glucose and lipids. Under conditions of obesity and nonalcoholic fatty liver disease (NAFLD), chronic supplementation of BCAAs enhances hepatic gluconeogenesis while inhibiting adipogenesis, and weakens insulin signaling through the mTORC1/2‐Akt2 cascade, leading to systemic substrate allocation [221]. In NAFLD models, elevated BCAA levels upregulate genes governing hepatic fatty acid oxidation yet fail to relieve insulin‐mediated lipogenic suppression, uncoupling oxidative metabolic reprogramming from IR phenotypes [222]. By contrast, moderate BCAA‐catabolic deficiency in lean mice improves glucose tolerance, enhances carbohydrate utilization, and remodels intrahepatic glycolytic, TCA cycle, and gluconeogenic networks, highlighting the context‐specific regulatory role of BCAA‐derived carbon and nitrogen flux in substrate selection [222]. BCAA intermediates also directly alter liver metabolism by inhibiting mitochondrial pyruvate carriers to suppress pyruvate‐driven gluconeogenesis, while increasing the production of lactate and β‐hydroxybutyrate, shifting cellular glucose metabolism toward lactate fermentation and fatty acid oxidation [223].

Multiple amino acid subclasses govern hepatic lipid synthesis, bile acid metabolism, and adipocyte programming. Excess dietary BCAAs elevate hepatic cytochrome P450 family 7 subfamily A member 1 (CYP7A1) and downstream bile acid synthesis by inhibiting the fibroblast growth factor 21‐extracellular regulated protein kinases pathway, which relieves endogenous suppression on CYP7A1 and establishes a direct regulatory link between BCAA metabolism and cholesterol‐bile acid homeostasis [224]. Aromatic amino acid supplementation under high‐fat dietary conditions suppresses intestinal farnesoid X receptor signaling, upregulates hepatic CYP7A1 and cytochrome P450 family 7 subfamily B member 1 expression, accelerates bile acid synthesis, and exacerbates adiposity, hepatic injury, and glucose intolerance [225]. Dietary BCAA restriction reverses IR and reduces pancreatic lipid accumulation in high‐fat fed mice via AMPK activation and mTORC1 inhibition, which rebalances de novo lipogenesis and fatty acid oxidation [226]. In insulin‐resistant obese rats, intensive nitrogen flux from BCAA metabolism consumes systemic Gly pools to support pyruvate–Ala cycling and Gln synthesis, triggering systemic Gly depletion and dysregulated triglyceride metabolism, which further validates profound interconnection between protein and lipid metabolic pathways [227].

Disruption of the integrated amino acid–glucose–lipid metabolism drives the onset and progression of systemic metabolic disorders. Acute elevation of interleukin‐6 (IL‐6) amplifies hepatic gluconeogenesis associated with Ala, pyruvate, and Gln, while inducing peripheral IR, which disrupts the coordinated carbohydrate and amino acid metabolic homeostasis by promoting amino acid‐to‐glucose conversion [228]. Dual acyl‐protein thioesterase 1/2 knockout mice present fasting hyperglycemia and IR accompanied by hyperactive Gln gluconeogenesis, demonstrating that fatty acid palmitoylation modifications directly couple lipid metabolic signaling to amino acid and glucose homeostasis via mitochondrial function and Gln transporter regulation including solute carrier family 1 member 5 (SLC1A5) [229]. Environmental microplastic exposure disrupts hepatic lipid accumulation, glycogenolysis, and the glucose transporter type 4‐AMPK axis alongside broad amino acid metabolic reprogramming, illustrating a complete pathological cascade where nutritional and environmental stressors provoke combined glucose and lipid metabolic dysfunction via amino acid metabolic perturbation [230].

In general, Ala and Gln maintain gluconeogenesis and blood glucose stability under fasting, diabetes and liver regeneration, and integrate muscle, liver and kidney metabolism into a continuous regulatory loop. Aromatic amino acids, BCAAs, and Ser‐Gly axis metabolites regulate metabolism through the TCA cycle replenishment, insulin‐signaling regulation, and substrate redistribution. When these interrelated regulatory circuits are damaged by inflammation, genetic or environmental damage, dysregulated substrates emerge, including excessive gluconeogenesis, heterotopic lipid deposition and systemic amino acid depletion, thus promoting the pathogenesis of T2D, NAFLD and peripheral nerve metabolic complications.

### 4.4 Gut Microbiota–Amino Acid Axis

Intestinal commensal microbes remodel host amino acid metabolic profiles by utilizing dietary and host‐derived nitrogenous substrates. Metabolites from microbial amino acid processing support intestinal epithelial energy supply and modulate intestinal barrier integrity, immune homeostasis, systemic metabolism, and neurological function via canonical signaling pathways. Cumulative evidence validates this regulatory framework, with refined mechanistic insights across diverse physiological and pathological contexts.

Gut microbes sustain systemic amino acid homeostasis by supplementing host EAA supply under nutritional limitation. Stable isotope tracing in mice confirmed microbial conversion of dietary carbohydrates into host‐available EAAs, especially valine (Val). Under low‐protein diets, microbial Val contribution to muscular amino acid pools can reach 60%, providing nutritional compensation for dietary protein insufficiency [231]. Reconstituted mouse microbiome studies further show that microbial EAA synthetic capacity depends on community structure, host physiology, and experimental conditions [232]. Microbiota perturbation can therefore reshape systemic amino acid availability and induce a BCAA‐deficiency phenotype in mammals [233].

Microbial amino acid metabolites govern intestinal mucosal homeostasis and local immune responses. BCAA‐derived metabolites including isovaleric acid alleviate oxidative stress, inflammation, and metabolic dysfunction in aging mice, via modulation of microbial ecology and host intracellular signaling cascades such as PI3K‐Akt and FoxO pathways [234]. β‐Glucan intervention mitigates mucosal injury in ulcerative colitis models, accompanied by microbial remodeling and elevated intestinal Trp and aromatic amino acid levels, highlighting amino acid metabolism as a core mucosal protective mediator [235]. *Lactobacillus acidophilus* supplementation alleviates hypertension‐induced intestinal barrier damage via Trp metabolic reprogramming, with 5‐hydroxyindoleacetic acid identified as a strong biomarker for mucosal injury and repair [236].

Microbial amino acid metabolism exerts broad regulatory effects on multiorgan systemic metabolism. Single‐strain screening and gene knockout models demonstrated that microbial BCAA‐ and Trp‐metabolic genes alter circulating amino acid profiles and modulate host glycemic homeostasis via peripheral serotonin signaling [89]. High‐protein diets reshape gut microbial communities, upregulate secondary bile acid synthesis and glucagon secretion, and enhance hepatic amino acid catabolism to construct a sequential gut–liver regulatory cascade [237]. Specific plant flavonoids ameliorate high‐fat diet‐induced metabolic dysregulation via restoring microbial homeostasis and correcting abnormal BCAAs and aromatic amino acid metabolism [238, 239]. In aged diabetic nephropathy mice, herbal intervention rectifies disordered Trp and Arg metabolism via microbial modulation, alleviating renal inflammatory damage and functional impairment [240].

This axis also mediates host aging, autoimmune susceptibility and neurological regulation. With advancing age, murine circulating Trp and its microbial metabolites decline, while proinflammatory Kyn accumulates, indicating a pathological shift in Trp metabolic partitioning [241]. In lupus‐prone mice, microbial dysbiosis drives excessive Kyn accumulation. Dietary Trp intake bidirectionally modulates autoimmune phenotypes and microbial colonization susceptibility, highlighting the central role of the microbiota–Trp axis in immune predisposition [242]. In Parkinson's disease models, specific probiotics elevate systemic BCAA levels and improve dopaminergic function and motor performance, confirming gut–brain axis neuroprotection via microbial BCAA metabolism [243]. Carbon isotope tracing further verified interorgan transport of microbial carbon into hepatic choline and cerebral Gln/GABA metabolism, expanding the scope of microbial metabolite systemic regulation [244].

Extrinsic microbial disruption can impair host–microbiota amino acid‐axis homeostasis. Broad‐spectrum antibiotics in mice reduce intestinal short‐chain fatty acids (SCFAs) and exert sex‐specific suppression on colonic EAAs, BCAAs, and aromatic amino acids, decoupling microbial and host metabolic correlations [245]. Similar patterns of amino acid disruption have been observed after microbiota depletion, supporting a role for microbial communities in maintaining host amino acid balance [246].

**FIGURE 4:**
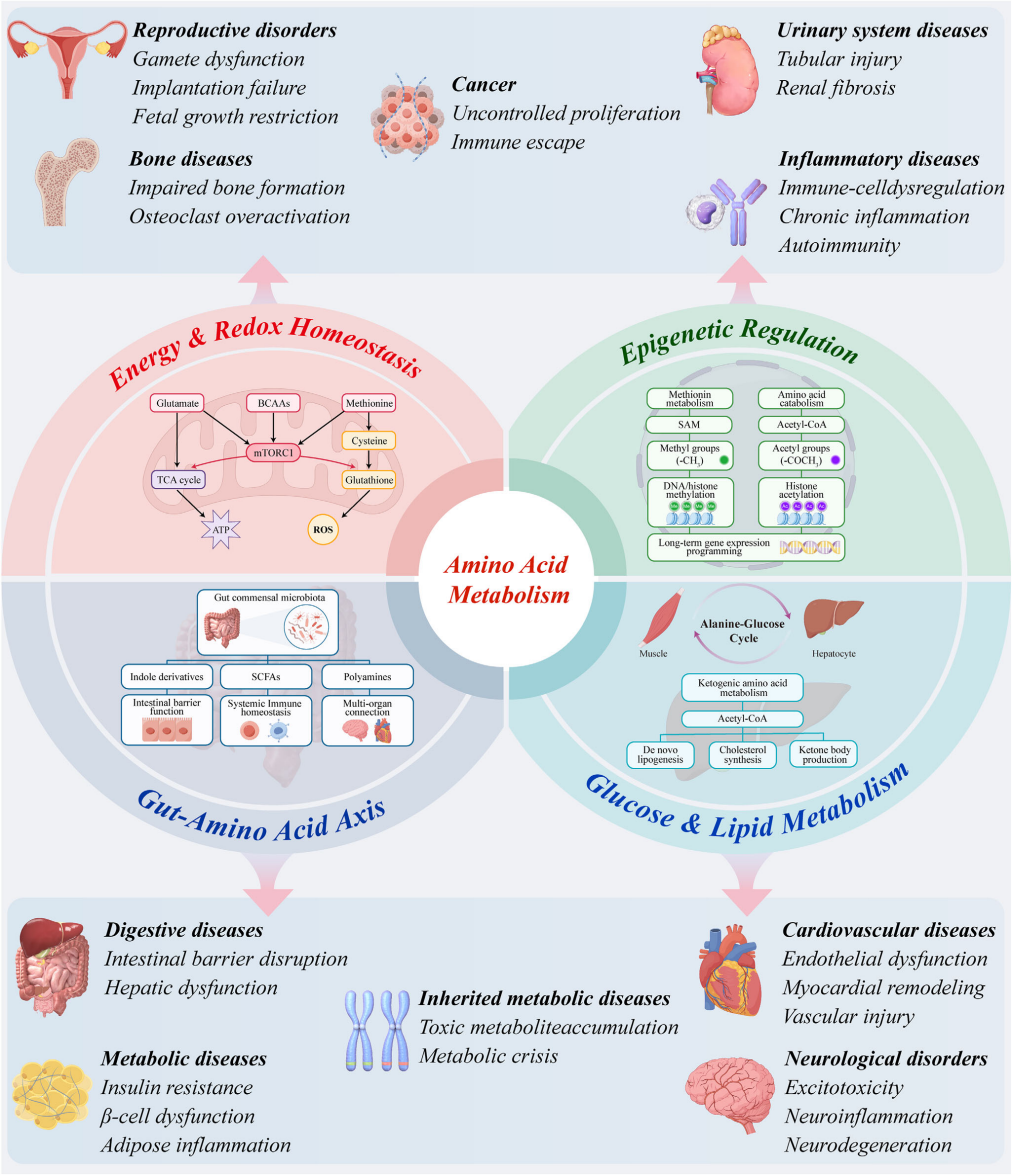
Core mechanistic hubs linking amino acid metabolism to systemic disease outcomes. This figure presents an integrative mechanism‐centered model in which amino acid metabolism connects energy and redox homeostasis, epigenetic regulation, the gut microbiota–amino acid axis, and glucose–lipid metabolic coordination to representative systemic disease outcomes. Acetyl‐CoA, acetyl‐coenzyme A; AhR, aryl hydrocarbon receptor; ATP, adenosine triphosphate; BCAAs, branched‐chain amino acids; mTORC1, mechanistic target of rapamycin complex 1; ROS, reactive oxygen species; SAM, S‐adenosyl methionine; SCFAs, short‐chain fatty acids; TCA, tricarboxylic acid.

Gut microbes utilize nonabsorptive dietary and host nitrogen substrates to drive targeted amino acid biosynthesis and catabolism. The resultant metabolites spanning BCAA derivatives, Trp catabolites, SCFAs, and polyamines sustain intestinal energy supply and host nutritional supplementation. These products modulate barrier maintenance, immune stability, insulin sensitivity, hepatic metabolism, neurological function and autoimmune tolerance via conserved signaling pathways, forming an integrated interactive regulatory system across microbes, amino acids and host physiology (Figure 4).

These mechanisms suggest a unifying model in which amino acid metabolism connects four homeostatic layers: bioenergetic/redox balance, chromatin and RNA modification, glucose–lipid coordination, and microbiota‐derived signaling. The main controversy is not whether these layers interact, but which layer is dominant in a given disease state. Disentangling this hierarchy will require models that perturb one hub at a time while monitoring compensatory responses across the other three hubs.

## 5 Dysregulation of Amino Acid Metabolism in Diseases

This section interprets disease through pathways rather than through organ lists alone. The subsections are organized by organ system, but they are compared through recurring pathogenic hubs: Glu–Gln–GABA imbalance in neural injury, Arg–NO failure in vascular disease, BCAA and aromatic amino acid signatures in IR, Trp–Kyn immune regulation, transporter defects in inherited disorders, and microbiota‐derived indole or polyamine signaling in inflammatory and renal disease. For each disease group, the central question is whether amino acid dysregulation acts as a driver, an adaptation, or a biomarker.

### 5.1 Neurological Disorders

The nervous system depends on tightly controlled amino acid metabolism to maintain excitation–inhibition balance, energy supply, and neuroinflammatory restraint. Disruption of these pathways creates shared metabolic vulnerabilities across neurodegenerative, psychiatric, and acute brain injury conditions.

The Glu–Gln–GABA cycle is the most fundamental amino acid metabolic pathway governing synaptic transmission and neural homeostasis. The stability of Glu, GABA, and Gln pools is essential for physiological synaptic transmission. Preclinical models have demonstrated that impairment of Gln synthetase or disruption of the Glu–Gln cycle triggers depressive‐like behaviors, epileptic seizures, and demyelination‐related neural damage [246, 247]. In experimental models of autoimmune encephalomyelitis and epilepsy, altered activity of enzymes and transporters involved in the Glu–Gln–GABA axis is highly correlated with excitotoxicity and disease‐related behavioral phenotypes. Notably, interventions such as N‐methyl‐D‐aspartate (NMDA) receptor antagonists or modulators of Glu catabolism can partially reverse these pathological alterations [248, 249].

Dysregulation of amino acid metabolism presents consistent yet disease‐specific patterns across distinct neurological disorders. In Alzheimer's disease (AD) models, early reprogramming of multiple amino acid metabolic pathways has been identified, including altered catabolism of BCAAs, disrupted Glu/GABA homeostasis, and dysregulated metabolism of Arg, Pro, and purine. These metabolic changes are associated with cognitive decline and can serve as early biomarkers for AD [250, 251]. For inflammation‐associated depression, clinical and preclinical studies have revealed activation of the Kyn pathway and elevated cerebral Glu levels, which drive neuroinflammation, excitotoxicity, and depressive‐like behaviors [252, 253]. In amyotrophic lateral sclerosis, spinal muscular atrophy, and HIV‐associated neurocognitive disorder, disrupted Glu–Gln balance, abnormal D/L‐amino acid homeostasis, and impaired Glu transport are core metabolic features, which contribute to progressive degeneration of motor neurons and spinal motor units [246, 254, 255]. Epilepsy is characterized by impaired Glu catabolism, altered GABA metabolism, and dysregulated activity of vitamin‐dependent enzymes, which disrupt the physiological balance between excitatory and inhibitory neurotransmission, leading to neuronal hyperexcitability and aggravated seizure activity [248].

The Trp–Kyn pathway has been identified as a shared mechanistic link across mood disorders, cognitive impairment, and neurodegenerative diseases. Multiple studies have demonstrated that a shift of Trp catabolism toward neurotoxic metabolites such as quinolinic acid is a common mechanism underlying depression, AD, Parkinson's disease, and autistic‐like behaviors. Neurotoxic Kyn metabolites exacerbate neuroinflammation by activating NMDA receptors, enhancing Glu excitotoxicity, and promoting oxidative stress [256, 257]. Interventions targeting this pathway, including IDO1 inhibitors, microbiota modulation, and mesenchymal stem cell therapy, have been shown to reduce quinolinic acid levels, regulate NMDA receptor subunit composition, and restore Glu transport, and alleviate depressive‐like behaviors and neuroinflammation in preclinical models [249, 258].

### 5.2 Cardiovascular Diseases

Under physiological conditions, the cardiovascular system depends on tightly regulated amino acid metabolism to maintain vascular integrity, myocardial energetic balance, and circulatory stability. Dysregulation of these pathways contributes to the onset and progression of multiple cardiovascular diseases, with aberrant metabolic processes triggering vascular injury, myocardial remodeling, and progressive cardiac functional decline.

Epidemiological cohort analyses have consistently linked elevated circulating Hcy to higher atherosclerotic plaque formation and hypertension risk [259, 260]. Excess Hcy promotes endothelial oxidative damage, vascular smooth muscle proliferation, and extracellular matrix remodeling, counteracting physiological vascular protective effects [261, 262, 263, 264]. Patient and in vitro investigations have also identified impaired Arg–NO signaling in hypertensive and atherosclerotic individuals, with reduced circulating Arg availability and endothelial NO synthase uncoupling diminishing NO production. Loss of this pathway's physiological vasodilatory and barrier‐preserving effects underlies endothelial dysfunction, the initiating event of atherosclerotic vascular disease and sustained blood pressure elevation [265, 266]. Preclinical data have further linked disrupted BCAA catabolism to atherosclerotic progression, with elevated circulating levels correlating with increased plaque lipid deposition and inflammatory macrophage activation [267, 268]. Dysregulated amino acid metabolism also disrupts physiological coagulation–anticoagulation balance, promoting a prothrombotic state in advanced atherosclerotic lesions [268].

In the setting of myocardial ischemia and heart failure, dysregulated amino acid metabolism mediates progressive myocardial dysfunction and adverse cardiac remodeling. BCAA catabolic pathways supporting physiological myocardial energy supply are impaired in failing hearts, with reduced BCKDH complex activity leading to intracellular accumulation of these amino acids and their toxic metabolites [269]. Cohort analyses have documented elevated myocardial and circulating BCAA levels in heart failure patients, with the degree of metabolic derangement correlating with worsening cardiac functional class [270]. Studies have verified that this impairment disrupts myocardial energetic homeostasis, alters intracellular calcium handling and promotes cardiomyocyte hypertrophy and fibrosis, underlying adverse ventricular remodeling and functional decline [271, 272]. Consistent alterations in Glu and Asp metabolism have been detected in ischemic myocardium, with impaired malate–Asp shuttle activity reducing mitochondrial oxidative capacity and exacerbating ischemic injury [273, 274].

In pulmonary arterial hypertension, impaired Arg–NO signaling reduces pulmonary vasodilatory capacity and drives pathological pulmonary vascular remodeling, mirroring findings in systemic hypertension [275]. Disrupted BCAA metabolism has also been implicated in the pathogenesis of cardiac arrhythmias, with accumulation of BCAA metabolites altering cardiomyocyte electrophysiological stability and increasing susceptibility to arrhythmic events [276].

These findings support a causal role for selected amino acid pathways in cardiovascular disease, particularly Hcy toxicity, Arg–NO dysfunction, and impaired BCAA catabolism. The strength of evidence varies across conditions, but the pathway links provide plausible targets for risk stratification and therapeutic intervention.

### 5.3 Digestive Diseases

Amino acid metabolic pathways sustain intestinal barrier integrity, digestive gland function and host–microbe symbiosis in the digestive system. Dysregulation of these pathways mediates the onset and progression of diverse digestive disorders, with aberrant metabolic processes triggering mucosal injury, inflammatory activation, and organ functional decline.

Cohort analyses of individuals with Crohn's disease and ulcerative colitis have consistently found reduced mucosal and circulating Gln and Arg, two amino acids supporting epithelial renewal and tight junction assembly under physiological conditions [277, 278]. Depletion of these amino acids underlies impaired intestinal barrier integrity, increased paracellular antigen translocation, and amplified mucosal inflammatory responses [279, 280, 281]. Preclinical models have identified disruptions in the gut microbiota‐amino acid axis, reduced bacterial conversion of Trp to indole derivatives, and impaired intestinal mucosal structure and function [282]. Loss of this physiological homeostatic signaling exacerbates inflammatory tone and visceral hypersensitivity, with similar metabolic changes detected in subsets of patients with irritable bowel syndrome [283].

The ratio of branched‐chain and aromatic amino acids is reduced in patients with cirrhosis and acute liver failure, which is a widely used indicator in clinical practice to assess the severity of the disease and the risk of hepatic encephalopathy [284, 285]. This ratio imbalance stems from impaired hepatic aromatic amino acid catabolism and reduced muscle BCAA uptake, promoting the generation of false neurotransmitters that disrupt cerebral function in hepatic encephalopathy [286]. Impaired hepatic urea cycle function is another consistent feature of advanced liver disease, leading to systemic hyperammonemia that exacerbates both hepatic injury and neurocognitive dysfunction [287, 288]. Dysregulated sulfur‐containing amino acid metabolism has also been detected in patients with NAFLD, with impaired GSH synthesis underlying hepatic oxidative stress and lipid accumulation [289].

In acute pancreatitis, rapid depletion of circulating and pancreatic Gln disrupts acinar cell metabolic homeostasis and promotes premature zymogen activation, thereby mediating pancreatic injury and systemic inflammatory responses [290]. Chronic pancreatitis is marked by progressive impairment of pancreatic exocrine function, with reduced digestive enzyme synthesis linked to altered amino acid availability and metabolic processes [291]. Amino acid metabolic reprogramming is also a well‐documented feature of gastrointestinal malignancies, with altered Gln and Ser catabolism supporting unregulated tumor cell proliferation and survival [292, 293].

Across digestive disorders, amino acid changes often map onto specific physiological failures, including barrier disruption, hepatic nitrogen intolerance, oxidative stress, and tumor metabolic rewiring. Some markers are already clinically useful, whereas others require causal validation before they can guide therapy.

### 5.4 Metabolic Diseases

Large‐scale epidemiological and genetic studies have established a causal association between elevated circulating BCAAs and IR as well as T2D. Notably, increased circulating BCAA levels can be detected 10–20 years prior to the clinical onset of diabetes, supporting their role as an early predictive marker [294, 295]. In high‐fat/high‐sucrose diet‐induced or genetic obesity models, the activity of BCAA‐catabolic enzymes such as BCKA dehydrogenase in adipose tissue and skeletal muscle is inhibited, leading to the accumulation of BCAAs and their BCKAs. This metabolic block is accompanied by systemic glucose and lipid metabolic disorders and elevated homeostatic model assessment for IR [296]. High BCAA intake or supplementation exacerbates obesity and IR by promoting proinflammatory M1 macrophage polarization and proinflammatory cytokine secretion in adipose tissue, which directly links impaired BCAA catabolism to adipose tissue inflammation and impaired insulin signaling [297, 298]. In addition, circulating aromatic amino acids are also elevated in the early stage of obesity, which is associated with a state of partially preserved insulin sensitivity but upregulated inflammatory response [299]. Preclinical studies have further validated that pharmacological activation of BCKDH via BCKDK inhibition reduces circulating BCAAs and BCKA levels, and significantly ameliorates IR in obese mice [300]. Interventions targeting gut microbiota to regulate BCAA production and catabolism, such as berberine and mulberry leaf extracts, also improve IR and T2D phenotypes via reducing circulating BCAA levels [301, 302, 303].

Long‐term reprogramming of multiple amino acid metabolic pathways is a consistent feature across all stages of T2D. Altered profiles of BCAAs, Gln, and Ala are widely detected in T2D patients. In high‐fructose diet or high‐fat diet combined with streptozotocin‐induced T2D models, early alterations in amino acid signatures can distinguish simple obesity from obesity with IR, indicating that these changes are early markers of metabolic imbalance and vascular risk [304]. For Ala metabolism, hepatic Ala aminotransferase 2 is upregulated in obesity and T2D, which enhances amino acid‐driven gluconeogenesis and acts as a driver of hyperglycemia. Correspondingly, knockdown of GPT2 reduces hyperglycemia in diabetic mice [216]. Circulating Ser and Gly levels are consistently reduced in metabolic syndrome and its complications including diabetic peripheral neuropathy. Inhibition of Ser synthesis or promotion of its catabolism accelerates the progression of diabetic neuropathy, while Ser supplementation partially reverses these pathological changes, highlighting the pathophysiological role of NEAAs in metabolic diseases and their complications [305].

Dysregulated amino acid metabolism also plays a mechanistic role in other common metabolic disorders. Purine synthesis and uric acid production, key pathological processes of hyperuricemia and gout, are closely associated with altered metabolism of Gly, Cys, and Gln, meaning amino acid profile changes can indicate uric acid load and gout flare risk [299]. Multiple animal and human cohort studies have demonstrated that aberrant amino acid metabolism, especially dysregulated BCAAs and aromatic amino acid pathways, frequently co‐occurs with NAFLD and cardiovascular complications. These metabolic alterations play a nodal role in adipogenesis, lipid oxidation, and systemic metabolic imbalance, supporting their potential as therapeutic targets for NAFLD [306].

Together, the evidence places BCAA and aromatic amino acid imbalance near the center of obesity, T2D, hyperuricemia, and NAFLD biology. These changes can promote insulin‐signaling defects, inflammation, altered gluconeogenesis, and lipid dysregulation, although their causal contribution depends on tissue context and disease stage.

### 5.5 Inflammatory and Immune Diseases

Amino acid metabolism is not a passive secondary alteration following inflammatory activation, but a regulatory node that determines immune cell fate, tissue microenvironment, and the intensity as well as duration of inflammatory responses. Dysregulation of these metabolic pathways is closely implicated in the pathogenesis of autoimmune diseases, inflammatory bowel disease (IBD), sepsis, allergic disorders, and immune senescence, with well‐characterized causal links between amino acid metabolic reprogramming and immune homeostasis disruption.

Gln and Arg depletion are core metabolic features of mucosal inflammation and autoimmune disorders. In preclinical models of ulcerative colitis, supplementation of Arg, Gln, or their combination with β‐hydroxy β‐methylbutyrate significantly reduces inflammatory markers including IL‐6, cyclooxygenase‐2, and myeloperoxidase, restores GSH and aldehyde dehydrogenase 4 family member A1 levels, and ameliorates mucosal structural damage. These findings demonstrate that these two amino acids are essential for antioxidant defense, mucosal barrier integrity, and epithelial repair [281, 307, 308]. Exogenous Gln supplementation also alleviates 5‐fluorouracil or lipopolysaccharide‐induced intestinal mucosal injury, via inhibiting toll‐like receptor 4/nuclear factor kappa‐B signaling, activating the nuclear factor erythroid 2‐related factor 2 antioxidant pathway, and restoring tight junction protein expression and gut microbiota homeostasis [281, 308]. Trp metabolism serves as another key regulatory axis for mucosal and systemic inflammation. Multiple studies have demonstrated that Trp catabolism via the indole/indole‐3‐acetic acid pathway activates AhR signaling, attenuates colitis in murine models, and restores intestinal barrier function, supporting Trp metabolic reprogramming as a promising therapeutic target for IBD [282, 309, 310].

Amino acid metabolism exerts precise regulatory effects on inflammatory responses via mediating metabolic–epigenetic crosstalk in macrophages and T cells. The nonproteinogenic amino acid α‐aminobutyric acid, generated from sulfur‐containing and small amino acid metabolism, inhibits proinflammatory M1 macrophage polarization, enhances mitochondrial OXPHOS, and boosts Gln/Arg metabolism. It also suppresses the expression of proinflammatory genes via increasing H3K27me3 modification, and alleviating inflammatory phenotypes in murine models of sepsis and colitis [213]. For T‐cell‐mediated autoimmunity, the long noncoding RNA LncBADR inhibits BCAA‐catabolic enzymes, leading to intracellular BCAA accumulation in T cells. This metabolic alteration activates the mTOR‐STAT1 signaling cascade, enhances interferon‐γ secretion, and exacerbates experimental autoimmune encephalomyelitis. Notably, knockout of LncBADR or restoration of BCAA catabolism significantly alleviates disease severity [311]. Dysregulation of BCAA‐catabolic enzymes BCAT1 and BCAT2 also drives inflammatory responses in diabetic retinopathy, psoriasis and obesity models, via altering α‐KG levels and histone methylation status to promote the expression of proinflammatory cytokines including IL‐6 and tumor necrosis factor‐α [147, 298, 312].

Dysregulated amino acid metabolism also plays a pivotal role in systemic inflammation, sepsis, immune senescence and autoimmune arthritis. Early sepsis metabolomic and amino acid kinetic evidence indicates that immune and metabolic stress can remodel amino acid handling before these changes are fully reflected in circulating profiles [147]. In aged mice, BCAAs and their SCFA metabolites reduce oxidative stress and systemic inflammation, and improve muscle and brain function, highlighting the potential protective role of the gut microbiota–amino acid axis in immune senescence [234]. For rheumatoid arthritis, a preclinical model showed that Zuo Gui Feng Teng Jian SH specifically inhibits Gln synthetase, downregulates Gln/Glu metabolism, corrects dysregulation of multiple amino acid pathways, and alleviates joint inflammation and synovial proliferation [313, 314]. In addition, reverse vaccine treatment for collagen‐induced arthritis partially restores the homeostasis of amino acid and carnitine pathways, indicating that concurrent metabolic and immune reprogramming is critical for disease remission [313].

Current evidence indicates that Arg, Gln, Trp, BCAAs, sulfur‐containing amino acids, and nonproteinogenic amino acids shape the initiation, amplification, and resolution of inflammation. They act through barrier repair, antioxidant defense, immune‐cell bioenergetics, and metabolic–epigenetic control. Dysregulation of these pathways can disturb effector‐regulatory balance and sustain tissue damage in autoimmune disease, IBD, psoriasis, sepsis, and immune senescence.

### 5.6 Bone Diseases

Amino acid metabolism is not merely a source of nutrients for skeletal tissue but a core metabolic hub that directly drives bone formation, resorption, and articular cartilage homeostasis. Multiple recent studies have validated the essential role of amino acid metabolism in skeletal health across osteoblasts, osteoclasts, hypothalamic‐sympathetic nerve regulation, and gut microbiota, with dysregulation of these pathways identified as a shared mechanistic node for diverse bone disorders.

Osteoporosis is characterized by amino acid metabolic dysregulation that directly impairs osteogenic and osteoclastic homeostasis. Metabolomic studies in ovariectomized osteoporosis mouse models have identified imbalances in Lys and Arg metabolism, which are closely associated with insufficient Type I collagen synthesis and reduced bone mineral density; supplementation of these amino acids effectively improves bone collagen metabolism and bone mass [315]. The Arg–NO pathway is another critical regulator of bone remodeling: L‐Arg supplementation promotes osteogenesis and angiogenesis, inhibits adipogenesis in ovariectomized mice, and significantly reduces bone loss via regulating mitochondrial autophagy and antioxidant defense [316]. In rheumatoid arthritis models, Arg supplementation also attenuates bone erosion via reprogramming osteoclast metabolism and inhibiting the receptor activator of nuclear factor‐κB ligand/RANK/TRAF6 signaling axis [317]. For osteoprogenitor cells, Gln catabolism via GLS1 provides carbon backbones and GSH, which are essential for nucleotide synthesis and antioxidant defense. Genetic deletion of GLS1 leads to impaired bone mineralization and osteogenesis, which can be partially rescued by supplementation of NEAAs and nucleotides [318]. The osteogenic lineage is also dependent on the transporter SLC38A2 for Pro and Ala uptake; genetic ablation of this transporter reduces osteoblast number and bone formation activity, resulting in low bone mass in vivo [319]. In addition, the L‐type amino acid transporter 1 (LAT1)–mTORC1 axis senses neutral amino acids in osteoclasts, and genetic deletion of LAT1 leads to excessive osteoclast activation and bone mass reduction [320].

Dysregulated amino acid metabolism also plays a pivotal role in osteoarthritis (OA) and cartilage metabolic remodeling. Following knee joint injury, subchondral bone osteocytes exhibit extensive reprogramming of amino acid and energy metabolism, including dysregulated pathways of Ala, Asp, Gln, Phe, and Tau. Altered BCAA profiles also emerge as potential biomarkers for OA, indicating that osteocyte metabolic imbalance precedes overt cartilage degeneration and drives OA progression [321]. A low‐molecular‐weight fish collagen heptapeptide protects chondrocytes both in vitro and in vivo, promotes the synthesis of Type I/II collagen and aggrecan, inhibits MMP‐3/‐13 expression and inflammatory/apoptotic signaling, and attenuates cartilage degeneration in OA models [322].

Amino acid metabolic pathways are also implicated in other bone‐related pathologies. Osteoclasts are dependent on Gln metabolism, with enhanced glutaminolysis as a metabolic hallmark of osteoclast differentiation and bone resorption. Genetic or pharmacological inhibition of this pathway significantly reduces osteoclast formation and ovariectomy‐induced bone loss [323]. Environmental Arg availability also determines the metabolic fate of multinucleated giant cells and osteoclast formation, and systemic restriction of Arg inhibits osteoclastogenesis and ameliorates bone damage in multiple mouse models of arthritis [324]. In addition, the LAT1‐mTORC1 axis in hypothalamic leptin receptor neurons senses amino acids and regulates sympathetic output; genetic ablation of this transporter leads to both energy imbalance and a high bone mass phenotype, linking central amino acid sensing to whole‐body bone homeostasis [325].

Collectively, amino acid supply, transport and catabolism are essential prerequisites for physiological bone remodeling. Reprogramming of specific amino acid pathways is a consistent feature of osteoporosis, OA and inflammatory/tumoral bone destruction. Targeted modulation of these pathways can effectively regulate bone mass and skeletal structure, supporting dysregulated amino acid metabolism as a shared pathogenic node and actionable therapeutic target for bone diseases.

### 5.7 Urinary System Diseases

The kidneys possess a specialized amino acid transport and metabolic system that maintains systemic nitrogen homeostasis and acid–base balance, supports the physiological functions of renal tubular epithelial cells, and contributes to the development of acute and chronic kidney injury, metabolic nephropathy, and genetic kidney disease; this system is not merely passively altered during disease progression.

In chronic kidney disease (CKD), extensive reprogramming of BCAAs and other amino acid metabolic pathways is a consistent pathological feature. In preclinical models of CKD and cardiorenal metabolic syndrome (CKM), impaired renal BCAA‐catabolic capacity leads to BCAA accumulation in the renal cortex, which is accompanied by aggravated proteinuria, renal hypertrophy, and pathological tissue damage. Pharmacological activation of BCAA catabolism via the BCKDK inhibitor BT2 reduces renal BCAA burden, ameliorates proteinuria, renal hypertrophy and pathological scores, and improves mitochondrial function, demonstrating that impaired BCAA metabolism is not only a biomarker, but also a causal driver and reversible therapeutic target for CKD and CKM [326]. In folic acid or adenine‐induced CKD models, metabolomic profiling revealed systematic alterations in energy metabolism, amino acid (including Gly) and organic osmolyte pathways along the progression from acute kidney injury (AKI) to CKD. These metabolic changes are consistent with the degree of renal fibrosis and functional deterioration, supporting the utility of amino acid signatures for disease stratification in CKD [327, 328]. In hyperuricemic kidney injury, elevated circulating levels of Ala, Ile, and Leu, coupled with reduced levels of Glu, Asp, Cys, and Gly, have been identified, which mediate the causal effect of hyperuricemia on CKD progression [329, 330].

In AKI, dysregulated amino acid transport and energy substrate depletion are closely linked to impaired renal repair and disease progression. Single‐cell RNA sequencing of renal tissues from a mouse model of ischemia–reperfusion AKI revealed that proximal tubular cells highly expressing neutral amino acid transporters (Slc6a19, Slc7a8, Slc43a2) are highly sensitive to ischemic injury, with their expression significantly decreased along with prolonged ischemia. Correspondingly, plasma Ile levels are markedly reduced, which has been validated in patients with cardiac surgery‐associated AKI, supporting its potential as an early biomarker for AKI [331]. In nephrotoxic AKI induced by aristolochic acid or cisplatin, BCAA catabolism and mitochondrial energy metabolism in proximal tubules are inhibited. Pharmacological activation of BCAA catabolism with BT2, or blockade of microRNA‐429‐3p‐mediated inhibition of the BCAA pathway, alleviates tubular necrosis, inflammatory responses and ferroptosis, and improves renal function [332, 333]. In sepsis‐induced AKI, significant alterations in renal cortical amino acids including Tau and Phe are closely associated with the progression of acute renal injury, indicating that dysregulated amino acid metabolism mediates the amplification loop of inflammation, oxidative stress, and energy disorder in AKI [334].

Dysregulated amino acid metabolism also acts as an upstream driver of metabolic and inherited kidney diseases. In diabetic kidney disease, multiple preclinical and clinical studies have identified significant dysregulation of renal BCAAs and TCA cycle‐related amino acids including Gln and Asp. Targeted modulation of these pathways, such as treatment with artemisinin derivatives or anthocyanins, simultaneously improves glycemic control, proteinuria and renal structural damage, validating amino acid metabolic alterations as both biomarkers and causal drivers of diabetic kidney disease [240, 335, 336, 337]. For hyperuricemia and uric acid nephropathy, Mendelian randomization analysis revealed that elevated uric acid levels specifically drive increases in circulating Ala and BCAAs, and Ala partially mediates the risk of uric acid‐induced chronic renal failure and elevated blood urea nitrogen. These findings indicate that targeted intervention of amino acids such as Ala may prevent uric acid‐related renal damage [329]. In adenine‐induced CKD models, dietary restriction of aromatic amino acids with normal protein intake reduces proteinuria, renal fibrosis, and inflammation to a similar extent as low‐protein diet, without increasing the risk of malnutrition. This renoprotective effect is mainly mediated by reducing the production of uremic toxins derived from aromatic amino acids, such as p‐cresol sulfate and indoxyl sulfate [338]. For inherited renal disorders, loss‐of‐function mutations in neutral amino acid transporters such as Slc6a19, which cause Hartnup disease, lead to impaired renal tubular amino acid reabsorption, aminoaciduria, and systemic nutritional and neurological complications, further validating the essential role of renal amino acid transport in systemic homeostasis [331].

Collectively, dysregulation of the renal amino acid transport and metabolic system is a consistent feature across CKD, AKI, uric acid nephropathy, and diabetic kidney disease. These disease‐associated amino acid metabolic alterations are closely linked to renal fibrosis, inflammation, and disease complications, and can serve as circulating or urinary biomarkers for early diagnosis and risk stratification. Meanwhile, these dysregulated pathways can be targeted through pharmacology, diet, or microbial interventions, supporting amino acid metabolism as a key regulatory node and promising therapeutic target for kidney disease.

### 5.8 Cancer

Amino acid metabolic reprogramming is a common feature of malignancy. Tumor cells alter uptake, catabolism, and biosynthesis to meet demands for biomass, energy, redox control, and immune evasion. These adaptations create therapeutic vulnerabilities, particularly when combined with chemotherapy, radiotherapy, or immunotherapy.

Dysregulated amino acid metabolism directly drives the acquisition and maintenance of malignant phenotypes via multiple conserved pathways. Gln addiction and aberrant amino acid transporter expression are well‐characterized features of colorectal cancer. In patient‐derived xenograft models of colorectal cancer, circulating levels of multiple amino acids including Phe, Asp and Met are significantly reduced, while the Gln transporters SLC1A5 and SLC7A5 are overexpressed in tumor tissues. These findings demonstrate that colorectal cancer cells are dependent on enhanced amino acid uptake and Gln‐fueled TCA cycle anaplerosis to support rapid proliferation and maintain redox homeostasis [339, 340]. For Ser and Gly metabolism, downregulation of ALDH1L1 leads to reprogramming of Ser/Gly‐related one‐carbon metabolism, antioxidant defense and fatty acid oxidation, which supports macromolecular synthesis and antioxidant defense in tumor cells [341]. In colorectal cancer, downregulated BCAA‐catabolic enzymes including ALDH2 and HCDH cause BCAA accumulation in tumor tissues, which is associated with poor overall survival of patients, supporting dysregulated BCAA catabolism as a prognostic marker and therapeutic target for colorectal cancer [342]. Methionine metabolism also plays a critical role in malignant progression: Met‐derived SAM regulates PCSK9 expression, mediates m6A/mRNA methylation, and drives immune evasion in multiple cancer types [214, 343].

Amino acid metabolic dysregulation is a central mediator of immunosuppression in the tumor microenvironment (TME). The Trp–IDO1–Kyn axis is a validated immune checkpoint pathway. Nanocarrier‐based delivery of the IDO1 inhibitor 1‐methyltryptophan reduces the intratumoral Kyn/Trp ratio, reverses local immunosuppression, and enhances the therapeutic efficacy of immune checkpoint blockade [344]. Arg depletion is another key mechanism of T‐cell suppression in cancer: tumor‐associated macrophages in pancreatic cancer highly express arginase 1, which degrades extracellular Arg and inhibits T‐cell activation. Genetic or pharmacological inhibition of Arg1 increases CD8+ T‐cell infiltration into the TME and enhances the antitumor effect of PD‐1 blockade [345]. In hepatocellular carcinoma, tumor cells compete with myeloid cells for Gln via SLC1A5, which triggers endoplasmic reticulum stress and G protein‐coupled receptor 109A upregulation in myeloid cells, driving the immunosuppressive polarization of myeloid‐derived suppressor cells and tumor‐associated macrophages. Blockade of G protein‐coupled receptor 109A or restoration of Gln availability restores the antitumor effector function of CD8+ T cells [339]. In addition, Met metabolism regulates immune checkpoint expression and immune evasion: Met‐derived SAM promotes m6A methylation and translation of PD‐L1 and V‐domain Ig suppressor of T‐cell activation, while dietary Met restriction or inhibition of the m6A reader YTH N6‐methyladenosine RNA‐binding protein 1 enhances CD8+ T‐cell infiltration and exerts a synergistic antitumor effect with PD‐1 inhibition [214, 343].

Dysregulated amino acid metabolism provides strong biomarkers and therapeutic targets for cancer management. In colorectal cancer patient‐derived xenograft models, 96 circulating metabolites dominated by amino acids have been identified as candidate diagnostic biomarkers, while SLC7A5 and SLC1A5 have been validated as promising therapeutic targets [340]. Alterations in serum or plasma amino acid profiles occur in a tumor progression‐specific manner across multiple cancer types, including breast cancer, T‐cell acute lymphoblastic leukemia, ovarian cancer and prostate cancer, supporting their utility for early cancer detection and disease progression monitoring [346, 347, 348]. For targeted therapy, the LAT1 inhibitor JPH203 restricts EAA uptake in triple‐negative breast cancer, induces amino acid starvation and cancer‐cell apoptosis, remodels the immunosuppressive TME, and enhances the therapeutic efficacy of PD‐1 antibodies [349].

Amino acid metabolic reprogramming not only supports tumor cell proliferation, nucleotide synthesis, and antioxidant defense, but also drives the formation of an immunosuppressive TME through depletion of Arg, Trp, and Gln, upregulation of amino acid transporters, and enhanced expression of immune checkpoints. Circulating and tissue amino acid characteristics can be used for early diagnosis and risk stratification of cancer, while targeted interventions against amino acid metabolism pathways are being tested in combination with chemotherapy, radiotherapy, and immunotherapy, demonstrating potential for enhancing antitumor efficacy and overcoming treatment resistance.

### 5.9 Inherited Metabolic Diseases

Inherited metabolic disorders related to amino acid metabolism arise from monogenic defects that disrupt specific steps of amino acid transport or catabolic pathways. These genetic lesions block physiological metabolic flux, trigger accumulation of toxic intermediate metabolites, and lead to highly characteristic clinical syndromes.

Maple syrup urine disease (MSUD) is a prototypical disorder of BCAA metabolism. It is caused by loss‐of‐function mutations in subunits of the BCKDH complex, including BCKDHA, BCKDHB, and DBT. The genetic defect impairs BCAA catabolism, leading to accumulation of BCAAs and their α‐keto acid derivatives in blood and tissues, which drives acute metabolic decompensation and severe neurotoxicity in affected neonates [349, 350]. Preclinical studies in MSUD mouse models have validated the causal link between enzyme deficiency, BCAA accumulation and disease pathogenesis. Delivery of the missing BCKDH subunit via lipid nanoparticle‐encapsulated mRNA restores BCKDH enzymatic activity, reduces circulating Leu levels, extends survival and improves body weight in affected mice, providing a proof‐of‐concept for mRNA‐based enzyme replacement therapy [351].

Additional BCAA‐related inherited disorders further define the physiological role of BCAA metabolic pathways. ECHS1 deficiency, caused by loss of short‐chain enoyl‐CoA hydratase, leads to severe neurodegeneration in affected patients [352]. Mechanistic studies indicate that Echs1 loss in neural stem and progenitor cells impairs neurogenesis through endoplasmic‐reticulum stress activation and lipid metabolic reprogramming [349]. A mouse model of ECHS1 deficiency further recapitulates neurological, metabolic, and inflammatory abnormalities, linking ECHS1 loss to disease‐relevant neurometabolic phenotypes [353]. Mucopolysaccharidosis Type IIIB, which arises from NAGLU deficiency and impaired glycosaminoglycan degradation, presents with secondary elevations in BCAAs and aromatic amino acids, together with dysregulated fatty acid metabolism in liver and heart. This finding demonstrates that a single enzymatic defect can perturb broader amino acid metabolic networks beyond the primary pathway [354]. Phenylketonuria, another classic inherited amino acid disorder, is caused by reduced Phe hydroxylase activity. The enzyme defect leads to accumulation of Phe and neurotoxic metabolites, resulting in severe neurodevelopmental impairment in untreated patients [355].

Genetic defects in amino acid transporters also cause systemic inherited disorders via impaired amino acid absorption or reabsorption. Mutations in the dibasic/cystine transporter SLC7A9 cause type B cystinuria. Spontaneous large‐fragment deletion of Slc7a9 in FVB/NJcl mice results in complete loss of transporter function, leading to high urinary cystine excretion and a high incidence of bladder stone formation. This model has been widely used as a strong tool to study the mechanisms of stone formation and test novel therapeutic interventions for cystinuria [356]. Loss‐of‐function mutations in the neutral amino acid transporter SLC6A19 cause Hartnup disease, which is characterized by impaired intestinal absorption of neutral amino acids. The resulting Trp deficiency reduces substrate availability for NAD synthesis, leading to pellagra‐like dermatitis and progressive neurological symptoms in affected patients. Studies in mouse models further show that heterozygous Slc6a19 deletion combined with gestational vitamin B3 deficiency causes NAD insufficiency, embryonic lethality and multiple developmental malformations, indicating that even nonenzymatic genetic lesions affecting amino acid transport can cause downstream cofactor deficiency and congenital defects via limiting amino acid availability [357].

Inherited amino acid disorders establish a genotype–metabolite–phenotype chain for amino acid pathway dysfunction. They also reveal therapeutic logic that is harder to infer from common diseases: reduce toxic substrate load, restore missing enzymatic activity, improve transport balance, or prevent downstream nitrogen and neurodevelopmental injury.

Recently characterized transporter and enzyme disorders extend this framework beyond classic phenylketonuria and MSUD. SLC7A7‐related lysinuric protein intolerance shows how impaired dibasic amino acid transport can cause a combination of intestinal malabsorption, renal aminoaciduria, urea‐cycle stress, pulmonary or renal complications, and skeletal phenotypes. Management is therefore moving from symptomatic protein control toward genotype‐aware monitoring, ammonia prevention, amino acid balancing, and enzyme‐ or transporter‐informed therapy. These rare disorders are not only pediatric diagnostic entities; they are natural models of systemic nitrogen, immune, skeletal, and renal homeostasis.

### 5.10 Reproductive System

Amino acids and their metabolic pathways regulate follicular development, endometrial receptivity, placental function, and sperm quality. In male reproduction, amino acid metabolism is a critical regulator of sperm function and fertilization capacity [358]. L‐amino acid oxidase 1 (LAO1) is specifically expressed in the sperm acrosome. Male mice with LAO1 deletion exhibit increased sperm malformation rate, reduced sperm motility, and decreased litter size in mating experiments. In vitro supplementation with an appropriate concentration of H2O2, a catalytic product of LAO1, restores the motility of sperm from LAO1 knockout mice, indicating that amino acid oxidation‐coupled redox signaling in sperm is essential for fertilization capacity. Further studies in bulls show that LAO1 content is significantly higher in sperm samples with high fertilization rate, supporting its potential as a biomarker for semen quality evaluation [359].

In female reproduction, dysregulated amino acid metabolism is closely implicated in ovarian dysfunction and reproductive disorders. In a mouse model of polycystic ovary syndrome induced by high‐fat and high‐sucrose diet, affected mice exhibit hyperandrogenism, ovulation disorder and impaired follicular development, accompanied by extensive dysregulation of ovarian amino acid metabolism, especially abnormal Arg biosynthesis. These metabolic alterations are consistent with the metabolic characteristics observed in human patients with polycystic ovary syndrome [360]. Supplementation with egg amino acids in animal models of limited nutrition increases circulating levels of multiple amino acids, enhances neutral amino acid transport in the ovary, elevates SAM and GSH levels, upregulates the expression of DNA methyltransferase and genes related to estrogen synthesis, and ultimately promotes follicular development and estrogen secretion, as well as increases the number of embryo implantation sites [361]. Trp metabolism also plays an irreplaceable role in the maintenance of endometrial receptivity and decidualization. Female mice fed with a Trp‐free diet show complete failure of embryo implantation, abnormal expression of markers for endometrial receptivity and decidualization, excessive activation of the IDO1–Kyn–AhR axis, and reduced 5‐hydroxytryptamine levels, demonstrating that dysregulated Trp metabolism directly disrupts the progression of early pregnancy [362].

During pregnancy, amino acid metabolism and transport are essential for placental angiogenesis, nutrient transfer and fetal growth. Livestock nutritional studies show that gestational micronutrient, sulfur, Arg, or N‐carbamylglutamate supplementation can alter placental amino acid transport, NO‐related pathways, antioxidant capacity, and fetal growth phenotypes [185, 363]. These observations are useful pathway‐level models, but their translational relevance should be framed around conserved placental transport, angiogenesis, NO, and mTORC1 signaling rather than species‐specific reproductive endpoints.

Maternal amino acid supply during pregnancy exerts long‐term programming effects on the health of offspring. Insufficient dietary protein and EAAs during pregnancy reduce circulating levels of EAAs, especially BCAAs, by 40%–50% in maternal mice. This nutritional deficiency leads to fetal growth restriction and alters mTOR signaling and neuronal differentiation in the hypothalamus of offspring, indicating that gestational amino acid deficiency increases the risk of metabolic disorders in offspring via central metabolic programming [364].

**FIGURE 5:**
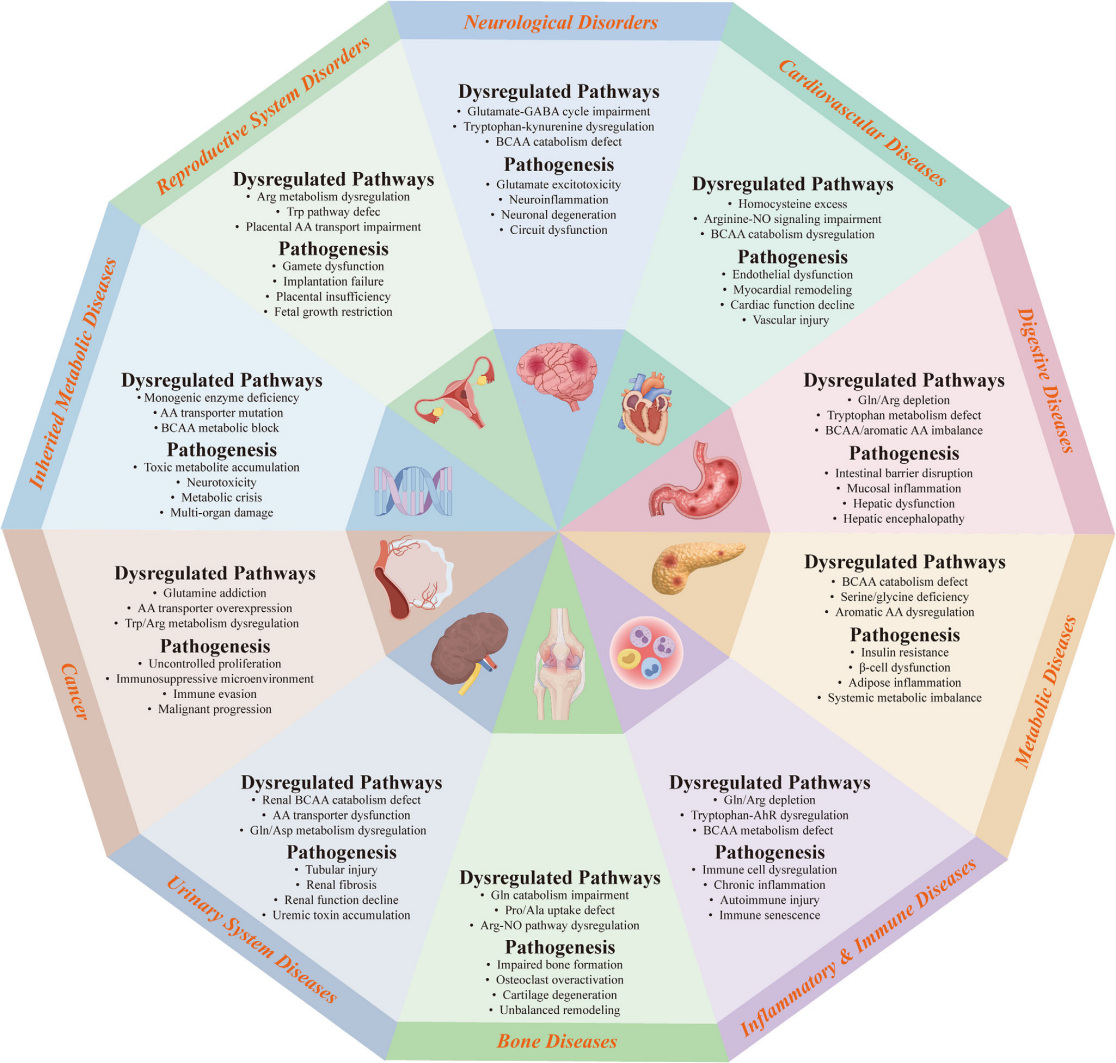
Dysregulated amino acid metabolism in the pathogenesis of human systemic diseases. This figure summarizes the core dysregulated pathways of amino acid metabolism and their corresponding pathogenic mechanisms across 10 major categories of human diseases, including neurological, cardiovascular, digestive, metabolic, inflammatory and immune, bone, urinary system, malignant, inherited metabolic, and reproductive system disorders, demonstrating that dysregulated amino acid metabolism is an active driver of disease initiation and progression rather than a secondary byproduct of pathological states. AA, amino acid; AhR, aryl hydrocarbon receptor; Ala, alanine; Arg, arginine; Asp, aspartate; BCAAs, branched‐chain amino acids; GABA, γ‐aminobutyric acid; Gln, glutamine; NO, nitric oxide; Pro, proline; Trp, tryptophan.

Amino acid metabolism contributes to spermatogenesis, follicular development, endometrial receptivity, placental angiogenesis, and nutrient transport. Its dysregulation can modify reproductive pathology and may provide targets for nutritional or metabolic intervention to improve fertility and pregnancy outcomes (Figure 5).

Several principles emerge across diseases. First, the same amino acid can be protective or pathogenic depending on tissue context, dose, and disease stage, as illustrated by BCAAs in metabolic, cardiovascular, and renal disorders. Second, circulating signatures often lag behind tissue‐level pathway activity, limiting causal interpretation. Third, inherited disorders provide the clearest gene‐to‐metabolite‐to‐phenotype evidence, whereas common diseases require more careful separation of primary drivers from adaptive responses. These distinctions explain why broad supplementation or restriction may fail without patient stratification.

## 6 Therapeutic Targeting of Amino Acid Metabolism

Clinical translation now spans enzyme replacement or depletion, nutrient restriction, transporter inhibition, immune‐metabolic modulation, and microbiota‐based strategies. Representative trials show both promise and limitation: ADI‐PEG20 in advanced hepatocellular carcinoma (NCT01287585, Phase 3; overall survival endpoint) did not improve survival; epacadostat plus pembrolizumab in melanoma (NCT02752074, Phase 3; progression‐free and overall survival endpoints) failed to improve outcomes; and telaglenastat plus cabozantinib in renal cell carcinoma (NCT03428217, Phase 2; progression‐free survival endpoint) did not outperform cabozantinib alone. By contrast, pegzilarginase for arginase 1 deficiency (NCT03921541, Phase 3; plasma Arg and functional endpoints) met its metabolic endpoint, and nanvuranlat/JPH203 targeting LAT1 has advanced to a global Phase 3 study in previously treated advanced biliary tract cancer (NCT07265674). These examples suggest that monogenic enzyme defects and biomarker‐selected tumors are closest to clinical use, whereas broad metabolic‐rewiring strategies remain more speculative.

### 6.1 Targeted Therapy

Therapeutic intervention has focused on rate‐limiting enzymes and transporters in cardiovascular, oncological, neurological, and immune‐mediated disease. BCKDK inhibitors such as BT2 activate BCKDH and restore impaired BCAA catabolism, with benefits reported in heart failure, CKM syndrome, and diabetes‐related cardiorenal injury, alongside mitochondrial protection and reduced ROS [326, 365]. Modulators of IDO1, tryptophan 2,3‐dioxygenase, and kynurenine 3‐monooxygenase reshape the Trp–Kyn pathway: inhibitors such as RY103 aim to enhance antitumor immunity, whereas targeted IDO1 upregulation may promote tolerance in autoimmune and neurodegenerative settings [366, 367]. Arginase inhibitors, including L‐norvaline (L‐Nva), can restore Arg–NO signaling and improve endothelial function in experimental atherosclerosis, hypertension, and diabetic complications [368]. CBS/3‐mercaptopyruvate sulfurtransferase modulators and GLS1 inhibitors further broaden the therapeutic landscape for sulfur amino acid disorders and Gln‐addicted malignancies [369].

Amino acid transporters and metabolite‐related receptors have also become druggable targets. LAT1, alanine–serine–cysteine transporter 2 (ASCT2), and EAAT2 were once viewed mainly as nutrient channels, but are now being explored in tumors, inflammation, and metabolic disease. The LAT1 inhibitor JPH203 has antitumor activity in several malignancies, and LAT1 can also serve as a delivery vector [370, 371]. ASCT2 inhibition targets KRAS‐mutated solid tumors, whereas EAAT2 modulation may be useful in systemic inflammation and metabolic syndrome [372, 373]. Metabolite‐sensitive receptors provide another layer of immune and metabolic regulation [374].

### 6.2 Dietary and Nutritional Interventions

The amino acid restriction diet represents an effective nutritional intervention model, with preclinical and preliminary population‐based evidence covering aging, metabolic disorders, and oncology. Lifelong or mid‐onset BCAA restriction extends lifespan and improves insulin sensitivity in mice, with selective restriction of Ile and Val identified as the core active component [375, 376]. Restriction of total or specific EAAs also alleviates hepatic steatosis in NAFLD, improves metabolic profiles in obesity and T2D, and enhances energy expenditure via fibroblast growth factor 21 upregulation [377]. Methionine restriction shows potential in NAFLD and one‐carbon metabolism‐related disorders, while selective amino acid deprivation extends survival in preclinical models of metastatic colorectal cancer [19, 378]. Notably, the benefits of these diets are modified by sex, immune status, and disease subtype, requiring precise patient stratification in clinical application [379].

Amino acid supplementation interventions are primarily applied in defined nutritional deficiency or pathological settings. In defined low‐protein states, BCAA supplementation can partially improve growth and food intake, supporting context‐dependent use in nutritional deficiency settings [380]. Moderate BCAA supplementation may improve hepatic steatosis in preclinical NAFLD models, highlighting disease‐specific applicability [19]. However, excessive free Lys supplementation disrupts systemic amino acid homeostasis and elevates circulating markers of diabetes risk, underscoring the safety hazards of unregulated supplementation [381]. At present, targeted supplementation of Gln, Arg, and BCAAs is best restricted to defined populations, such as critically ill patients, patients with liver cirrhosis, and immunocompromised individuals, where clinical guideline evidence is available.

Dietary therapy is the cornerstone of clinical management for inherited amino acid metabolic disorders, with continuous advances in formula development and adjuvant therapeutic strategies. For classic phenylketonuria, the standard of care consists of a natural protein‐restricted diet, Phe‐free/low‐Phe synthetic amino acid formulations, and low‐protein specialized foods [382, 383]. Emerging strategies include large neutral amino acid supplementation, which maintains stable brain Phe levels with more lenient dietary restrictions, and novel low‐Phe protein formulations with improved palatability and long‐term adherence [384]. For MSUD, standard treatment relies on a lifelong low‐BCAA diet, with novel adjuvant approaches including mRNA‐based enzyme replacement therapy and oral engineered Leu decarboxylase to control circulating BCAA levels [349, 385]. Comprehensive management also requires consideration of central nervous system targeting to avoid irreversible neurodevelopmental impairment [386].

Dietary and nutritional interventions remain the most established way to modify amino acid metabolism in the clinic. Their value is clearest in inherited disorders and defined nutritional states, while broader use in complex diseases will depend on patient selection, monitoring, and long‐term safety.

### 6.3 Small‐Molecule Regulators

Approved or clinically applied amino acid‐targeted agents are dominated by engineered enzyme preparations. PEGylated L‐asparaginase depletes circulating asparagine and Gln and is approved for first‐line treatment of acute lymphoblastic leukemia; preclinical work has also explored senolytic effects in aging‐related disorders such as atherosclerosis, osteoporosis, and NAFLD [387, 388]. Pegzilarginase, a PEGylated arginase, depletes systemic Arg and has been developed for ASS1‐deficient solid tumors, with safety and combination activity supported in patient‐derived xenograft models [389]. The combination of ADI‐PEG20 with the arginase inhibitor L‐Nva represents a bidirectional Arg‐metabolism strategy with preclinical efficacy in melanoma models [390].

Many small‐molecule regulators remain in preclinical or early clinical development. Arginase inhibitors, including L‐Nva and NG‐hydroxy‐nor‐L‐Arg, have shown activity in experimental breast cancer, hypertension, and diabetic complications, and 18F‐labeled arginase inhibitor probes may help patient stratification [391, 392, 393]. LAT1‐targeted inhibitors and delivery systems are also advancing; JPH203 shows antitumor activity and synergy with PD‐1 antibodies in triple‐negative breast cancer models, while LAT1‐mediated delivery can target the brain, pancreas, and tumor tissues [394]. Targeting the cystine/Glu antiporter with silymarin may also improve metabolic dysfunction‐associated fatty liver disease by regulating Cys metabolism and ferroptosis [395].

These agents support the therapeutic value of amino acid pathways, but they also highlight the need for specificity, pharmacodynamic biomarkers, and tissue‐targeted delivery. The next generation of modulators will need to widen the therapeutic window rather than simply intensify pathway inhibition.

### 6.4 Microbiota‐Based Strategies

Probiotics and prebiotics are the most widely studied interventions for the microbiota–amino acid axis. Flavonoid and polysaccharide prebiotics can reshape gut microbiota, normalize circulating and fecal BCAAs, and remodel Trp metabolism, improving insulin sensitivity in high‐fat diet‐induced metabolic syndrome and T2D models [239, 396]. *Lactobacillus* strains increase Trp‐derived indole metabolites, activate antioxidant and anti‐inflammatory pathways, and alleviate IBD in preclinical models [397]. Other probiotic strategies modulate BCAA biosynthesis and Trp–indole metabolism in Parkinson's disease, AD, and anxiety‐ or depression‐like behavioral models [243, 398, 399].

Fecal microbiota transplantation (FMT) and engineered bacterial strains aim to rebuild favorable amino acid metabolic environments. Healthy‐donor or young‐donor FMT can transfer metabolic features linked to BCAAs, Trp, and polyamine metabolism in mammalian disease and aging models [400, 401]. Probiotic‐optimized FMT can increase immunomodulatory indole metabolites and improve colitis outcomes [402]. Engineered or donor‐transfer bacterial approaches that target indole–AhR or polyamine pathways remain largely preclinical and may be refined through donor selection, probiotic support, and metabolite‐focused design [403, 404].

Direct delivery of microbiota‐derived amino acid metabolites is another therapeutic route. Indole derivatives and polyamines can regulate inflammation, infection, neuroinflammation, and renal fibrosis through AhR signaling and related pathways. For example, indole‐3‐propionic acid and indole‐3‐acetic acid alleviate intestinal inflammation in preclinical colitis models, and colon‐targeted indole‐3‐propionic acid prodrugs may improve local efficacy [405]. Indole derivatives also reduce Aβ deposition and neuroinflammatory signaling in AD models, whereas polyamine‐related metabolic correction shows renoprotective effects in CKD models [406, 407].

**FIGURE 6:**
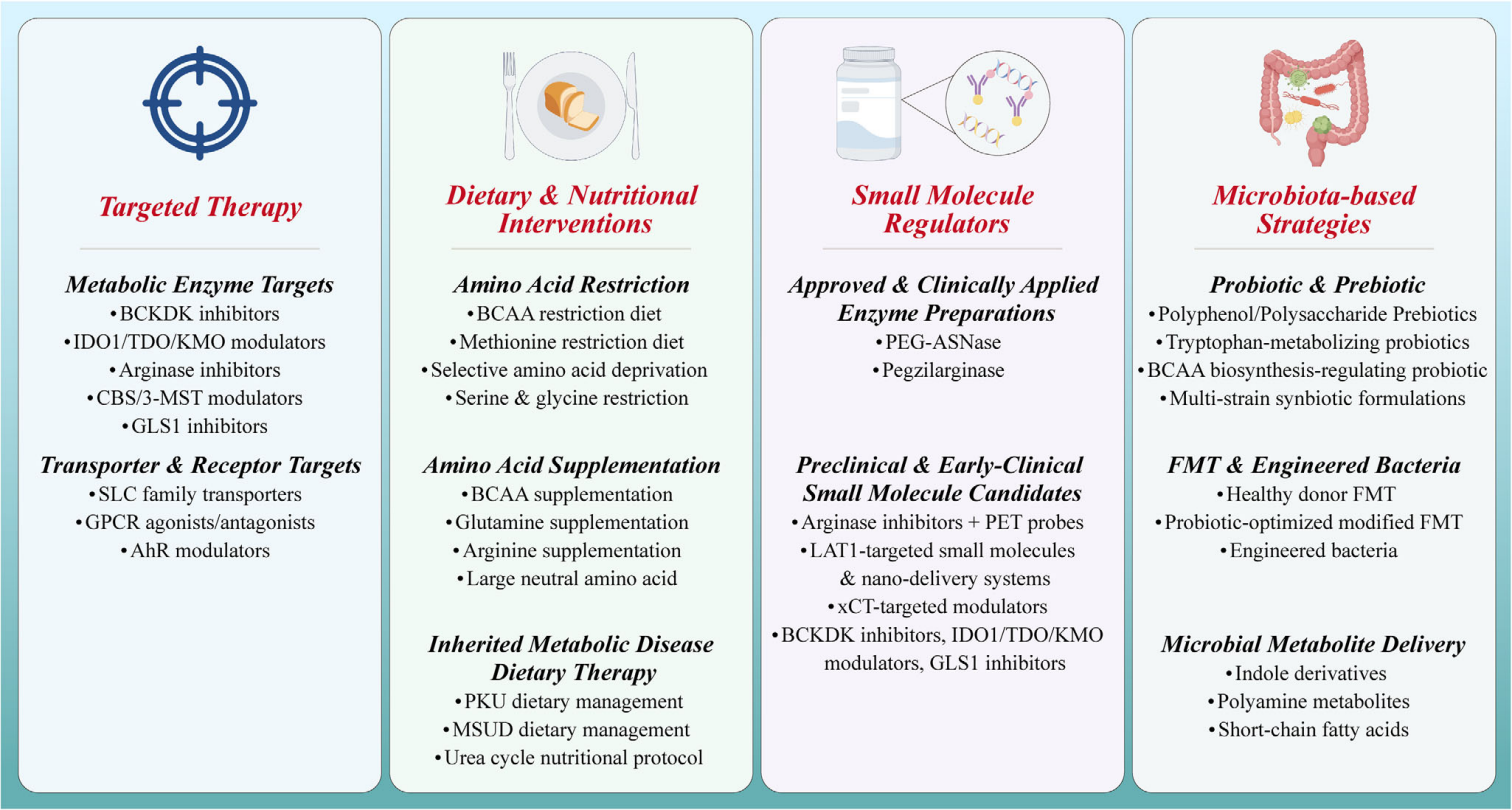
Therapeutic strategies targeting amino acid metabolism in human diseases. This figure systematically outlines four major categories of therapeutic strategies targeting amino acid metabolism, including targeted therapy against rate‐limiting enzymes and transporters, dietary and nutritional interventions, small‐molecule regulators, and microbiota‐based strategies, summarizing the core intervention approaches and key candidates for clinical application and preclinical development. AhR, aryl hydrocarbon receptor; BCAAs, branched‐chain amino acids; BCKDK, branched‐chain α‐keto acid dehydrogenase kinase; CBS, cystathionine β‐synthase; FMT, fecal microbiota transplantation; GLS1, glutaminase 1; GPCR, G protein‐coupled receptor; IDO1, indoleamine 2,3‐dioxygenase 1; KMO, kynurenine 3‐monooxygenase; LAT1, L‐type amino acid transporter 1; MSUD, maple syrup urine disease; PEG‐ASNase, pegylated L‐asparaginase; PET, positron emission tomography; PKU, phenylketonuria; SLC, solute carrier; TDO, tryptophan 2,3‐dioxygenase; 3‐MST, 3‐mercaptopyruvate sulfurtransferase; xCT, cystine/glutamate antiporter.

Clinical translation shows that target engagement alone is not enough. Failed trials point to recurring barriers: redundant nutrient acquisition, weak patient selection, adaptive pathway rewiring, inadequate pharmacodynamic biomarkers, and toxicity from disturbing amino acid pools shared by normal tissues. The strategies most likely to enter routine use within 5–10 years are enzyme replacement for defined inherited disorders, monitored dietary protocols, and transporter or enzyme inhibitors in biomarker‐selected tumors. Microbiota engineering, broad amino acid restriction for complex disease, and multitarget immunometabolic combinations remain promising but need stronger safety, durability, and stratification data (Figure 6).

## 7 Conclusions and Perspectives

Amino acid metabolism is no longer viewed only as a source of energy and protein building blocks. It is now recognized as a regulatory network that links epigenetic modification, signal transduction, immune homeostasis, redox balance, nitrogen handling, and organ‐specific physiology. Its dysregulation is reported across cancer, metabolic disease, cardiovascular disease, renal disease, immune‐mediated disorders, inherited metabolic syndromes, and reproductive pathology. The mechanisms differ by tissue, but they commonly involve malignant proliferation and immune escape in the TME, IR and inflammation in metabolic and cardiovascular disease, renal functional decline, and toxic metabolite accumulation in inborn errors of metabolism. Therapeutic strategies now include enzyme and transporter targeting, amino acid restriction or depletion, dietary protocols, microbiota‐based interventions, and combination therapy with immunotherapy or chemotherapy.

Several gaps still constrain interpretation and translation. Many studies rely on circulating amino acid profiles, whereas tissue‐specific and cell‐subtype‐specific activity, especially in the TME and immune compartments, remains poorly mapped. Host–microbe amino acid exchange also needs more direct mechanistic study, including how microbial metabolites affect distant organs along the gut–liver, gut–brain, and gut–kidney axes. Clinically, single‐center designs, small cohorts, population heterogeneity, limited biomarkers, and sparse long‐term safety data continue to slow therapeutic development.

Progress will require integrated approaches that combine spatial metabolomics, single‐cell transcriptomics, and isotope tracing to resolve pathway activity at cellular and tissue levels. Mechanistic studies should test how amino acid flux interacts with epigenetic regulation and microbial metabolite signaling rather than measuring each layer separately. Patient‐derived organoids, humanized microbiome models, and larger multicenter trials will be essential for bridging preclinical findings and clinical application. Future therapeutic development should prioritize tissue‐specific small molecules, localized metabolite delivery by engineered bacteria, personalized nutrition guided by host genotype and microbiome state, and rational multitarget combinations.

Cross‐disciplinary integration of metabolomics, computational biology, and clinical medicine will determine how far the field moves beyond association. The most useful framework will link amino acid classes, sensing pathways, organ‐specific flux, microbiota‐derived metabolites, and clinical phenotypes into testable models rather than independent lists. Such models can identify actionable targets and precision interventions for malignant, metabolic, inherited, immune‐mediated, and neurodegenerative disorders.

## Author Contributions

**Lianjun Xing and Tao Wu**: conceptualization, supervision, writing – review and editing. **Zhiwei Su and Jinyu Liu**: data curation, methodology, software, writing – original draft preparation. **Xiaoliang Deng, Yanqun Luo, Haiping Xue, and Lei Wang**: writing – review and editing. All the authors have approved the final manuscript.

## Funding

This work was supported by the National Natural Science Foundation of China (82574670), National Natural Science Foundation of Shanghai (24ZR1466600), Excellent Project of 2024 Shanghai Oriental Talents (BJWS2024050), Shanghai Municipal Science and Technology Commission Science and Technology Innovation Action Plan for Major and Intractable Diseases Research (24Y12801100), and Shanghai Hospital Development Center Foundation (SHDC22026219 and SHDC12026108).

## Ethics Statement

The authors have nothing to report.

## Conflicts of Interest

The authors declare no conflicts of interest.

## Data Availability

All data are freely available from the corresponding author upon request.
